# Epigenomic signature of accelerated ageing in progeroid Cockayne syndrome

**DOI:** 10.1111/acel.13959

**Published:** 2023-09-08

**Authors:** Clément Crochemore, Claudia Chica, Paolo Garagnani, Giovanna Lattanzi, Steve Horvath, Alain Sarasin, Claudio Franceschi, Maria Giulia Bacalini, Miria Ricchetti

**Affiliations:** ^1^ Institut Pasteur, Université Paris Cité, Molecular Mechanisms of Pathological and Physiological Ageing Unit, UMR3738 CNRS Paris France; ^2^ Institut Pasteur, Team Stability of Nuclear and Mitochondrial DNA, Stem Cells and Development, UMR3738 CNRS Paris France; ^3^ Sup'Biotech Villejuif France; ^4^ Institut Pasteur, Université Paris Cité, Bioinformatics and Biostatistics Hub Paris France; ^5^ IRCCS Azienda Ospedaliero‐Universitaria di Bologna Bologna Italy; ^6^ Department of Medical and Surgical Sciences (DIMEC) University of Bologna Bologna Italy; ^7^ CNR Institute of Molecular Genetics “Luigi Luca Cavalli‐Sforza”, Unit of Bologna Bologna Italy; ^8^ IRCCS Istituto Ortopedico Rizzoli Bologna Italy; ^9^ Department of Human Genetics, David Geffen School of Medicine University of California Los Angeles USA; ^10^ Department of Biostatistics Fielding School of Public Health University of California Los Angeles USA; ^11^ Laboratory of Genetic Stability and Oncogenesis, Institut de Cancérologie Gustave Roussy University Paris‐Sud Villejuif France; ^12^ Institute of Information Technologies, Mathematics and Mechanics Lobachevsky University Nizhniy Novgorod Russia; ^13^ IRCCS Istituto delle Scienze Neurologiche di Bologna Bologna Italy

**Keywords:** ageing, Cockayne syndrome, DNA methylation, epigenetic clock, progeroid diseases, UV‐sensitive syndrome

## Abstract

Cockayne syndrome (CS) and UV‐sensitive syndrome (UVSS) are rare genetic disorders caused by mutation of the DNA repair and multifunctional CSA or CSB protein, but only CS patients display a progeroid and neurodegenerative phenotype, providing a unique conceptual and experimental paradigm. As DNA methylation (DNAm) remodelling is a major ageing marker, we performed genome‐wide analysis of DNAm of fibroblasts from healthy, UVSS and CS individuals. Differential analysis highlighted a CS‐specific epigenomic signature (progeroid‐related; not present in UVSS) enriched in three categories: developmental transcription factors, ion/neurotransmitter membrane transporters and synaptic neuro‐developmental genes. A large fraction of CS‐specific DNAm changes were associated with expression changes in CS samples, including in previously reported *post‐mortem* cerebella. The progeroid phenotype of CS was further supported by epigenomic hallmarks of ageing: the prediction of DNAm of repetitive elements suggested an hypomethylation of *Alu* sequences in CS, and the epigenetic clock returned a marked increase in CS biological age respect to healthy and UVSS cells. The epigenomic remodelling of accelerated ageing in CS displayed both commonalities and differences with other progeroid diseases and regular ageing. CS shared DNAm changes with normal ageing more than other progeroid diseases do, and included genes functionally validated for regular ageing. Collectively, our results support the existence of an epigenomic basis of accelerated ageing in CS and unveil new genes and pathways that are potentially associated with the progeroid/degenerative phenotype.

## Graphical abstract

We studied DNA methylation (DNAm) in a unique human model where impairment of the same protein results or not in a progeroid disease. Functional analyses of DNAm of accelerated ageing reveal enrichment in developmental transcription factors, ion/neurotransmitter transporters, and synaptic neurodevelopmental genes. A large fraction of DNAm changes has been associated with expression changes in multiple patient samples. Progeroid Cockayne syndrome is a pertinent model also to study ageing.

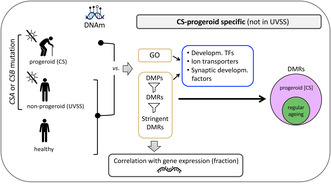

**Abbreviations**
CS: Cockayne syndrome
CSA: Cockayne syndrome group A protein
CSB: Cockayne syndrome group B protein
DMPs: differentially methylated positions
DMRs: differentially methylated regions
DNAm: DNA methylation
GO: Gene Ontology
TFs: transcription factors
UV: ultraviolet
UVSS: UV‐sensitive syndrome

## 1 INTRODUCTION

Progeroid syndromes are a group of rare genetic disorders in which ageing appears to be greatly accelerated (Burtner & Kennedy, 2010). These conditions are genetically, biochemically, and clinically heterogeneous, with the extent and combination of ageing‐related defects (Aryee et al., 2014) being specific to each progeroid syndrome. While the Hutchinson–Gilford progeria syndrome (HGPS) is due to a defective nuclear lamina as a consequence of mutations in *LMNA* gene, the Down syndrome (DS) is due to trisomy of chromosome 21. The Werner syndrome (WS) and Cockayne syndrome (CS) result from mutation in multifunctional proteins best known for their role in DNA repair (Rieckher et al., 2021).

Here, we focus on CS which is a devastating multisystemic disease characterized by neuro‐developmental defects, precocious ageing, and in most cases cutaneous photosensitivity. It is a progressive disorder with most symptoms appearing relatively early in life and worsening with time (Natale, 2011). Multiple degrees of severity have been described, including a prenatal‐onset form known as cerebro‐oculo‐facioskeletal (COFS) syndrome, an early‐onset severe form (CS‐II, average age of death 5–6 years), a classical or moderate form (CS‐I, mean life expectancy around 16 years), and a mild or late‐onset form (CS‐III) (Laugel et al., 2010).

Cockayne syndrome is due to mutations in either *CSB* or *CSA* genes (approximately 70:30 ratio) (Calmels et al., 2018; Laugel et al., 2010), which code repair factors of UV‐induced DNA damage through the transcription‐coupled nucleotide excision repair (TC‐NER) pathway (Mayne & Lehmann, 1982). CS defects, including the precocious ageing phenotype, have been essentially ascribed to impaired TC‐NER. Nevertheless, three patients carrying mutations in *CSB* or *CSA* developed an independent syndrome, the UV‐sensitive syndrome (UVSS) that is characterized by severe photosensitivity but no sign of premature ageing (Horibata et al., 2004; Nardo et al., 2009). Only a few UVSS patients, which display a common phenotype, are known worldwide (Nardo et al., 2009). Most UVSS cases are due to mutations in UVSSA, a protein that acts in TC‐NER in concert with CSA and CSB (Sarasin, 2012; van der Weegen et al., 2020). These case reports, at least those mutated in *CSB* or *CSA*, de facto uncouple the DNA repair defect from precocious ageing and suggest that impairment of other functions of multifunctional CSA and CSB proteins are responsible for progeroid defects.

Indeed, CSA and CSB are also transcription factors (Kristensen et al., 2013), CSB is implicated in chromatin remodelling (Citterio et al., 2000; Newman et al., 2006), and have been detected in mitochondria where they act in repairing oxidative damage (Kamenisch et al., 2010) and promoting transcription (Berquist et al., 2012) of mitochondrial DNA. Additionally, we showed that in CS (but not UVSS) cells, the CSA/CSB impairment results in overexpression of the HTRA3 protease leading to degradation of the mitochondrial DNA polymerase POLG1, and consequent mitochondrial dysfunction (Chatre et al., 2015). CSA and CSB mutations represent therefore an exceptional case for disentangling downstream genes and pathways involved in dramatically different phenotypes, since their impairment results (CS) or not (UVSS) in accelerated ageing. We recently associated the CS‐specific HTRA3/POLG1/mitochondrial alterations with replicative senescence in normal cells, and identified CSB depletion as an early trigger of this process, mechanistically linking progeroid defects with a major ageing‐related process (Crochemore et al., 2019).

Different molecular hallmarks of ageing (e.g., epigenetic alterations, senescence and mitochondrial dysfunction) have been identified (Lopez‐Otin et al., 2013). DNA methylation (DNAm), an epigenetic mechanism corresponding to the addition of a methyl group in the fifth position of a cytosine (Moore et al., 2013), is of particular relevance in the study of ageing and age‐related conditions, as it substantially contributes to genomic stability and can regulate age‐dependent gene expression (Ciccarone et al., 2018). DNAm globally decreases with ageing, largely as a consequence of demethylation of repetitive retroelements (LINEs, *Alu* sequences), driving genomic instability (Ciccarone et al., 2018). At the same time, age‐dependent hypermethylation or hypomethylation events have been reported for specific CpG sites located within genic sequences, although their functional consequences for gene expression changes are not straightforward (Yuan et al., 2015). Subsets of these specific sets of CpGs have been used to build epigenetic clocks, that is, predictors of chronological age that are also informative of biological age and mortality risk (Horvath, 2013; Levine et al., 2018; Lu et al., 2019).

Previous studies reported alterations of the DNAm profile in WS‐, HGPS‐ and DS‐derived cells compared to healthy controls (Guastafierro et al., 2017; Heyn et al., 2013; Horvath et al., 2015; Kohler et al., 2020) and discussed their possible contribution to the progeroid phenotype based on the comparison of changes occurring in physiological ageing. Interestingly, mild increased epigenetic age calculated using epigenetic clocks was reported for all the three conditions (Horvath et al., 2015; Horvath & Raj, 2018; Maierhofer et al., 2017). On the contrary, the epigenetic landscape of CS has not been described so far, and differences with non‐progeroid UVSS have not been addressed.

In the present study we performed genome‐wide analysis of the methylome of CS, UVSS and WT fibroblasts. We showed that large genome‐wide DNAm changes occur in mutated cells (UVSS and CS). In order to focus on the DNAm alterations that are associated with the age‐accelerated phenotype of CS, we took advantage of the availability of fibroblasts from two very rare UVSS patients, that are mutated but non‐progeroid. We thus included UVSS in the group of ‘Non‐Progeroid’ controls, together with WT cells, and compared them with CS cells. This analysis identified CpG sites mapping in genes enriched in three major functional categories, and, importantly, the most significant of them display a methylation/transcription correlation according to CS transcriptomic datasets. We also demonstrated a dramatic increase of the epigenetic age of CS fibroblasts, and comparison with available datasets revealed a CS epigenetic signature closer to physiological ageing than HGPS, WS or DS, underscoring CS as a relevant model of accelerated ageing. In support of this finding, we report that a majority of top differentially methylated genes have been functionally validated in age‐related processes.

## 2 RESULTS

### 2.1 Identification of differentially methylated positions/regions (DMPs/DMRs) associated with the CS progeroid phenotype

**FIGURE 1:**
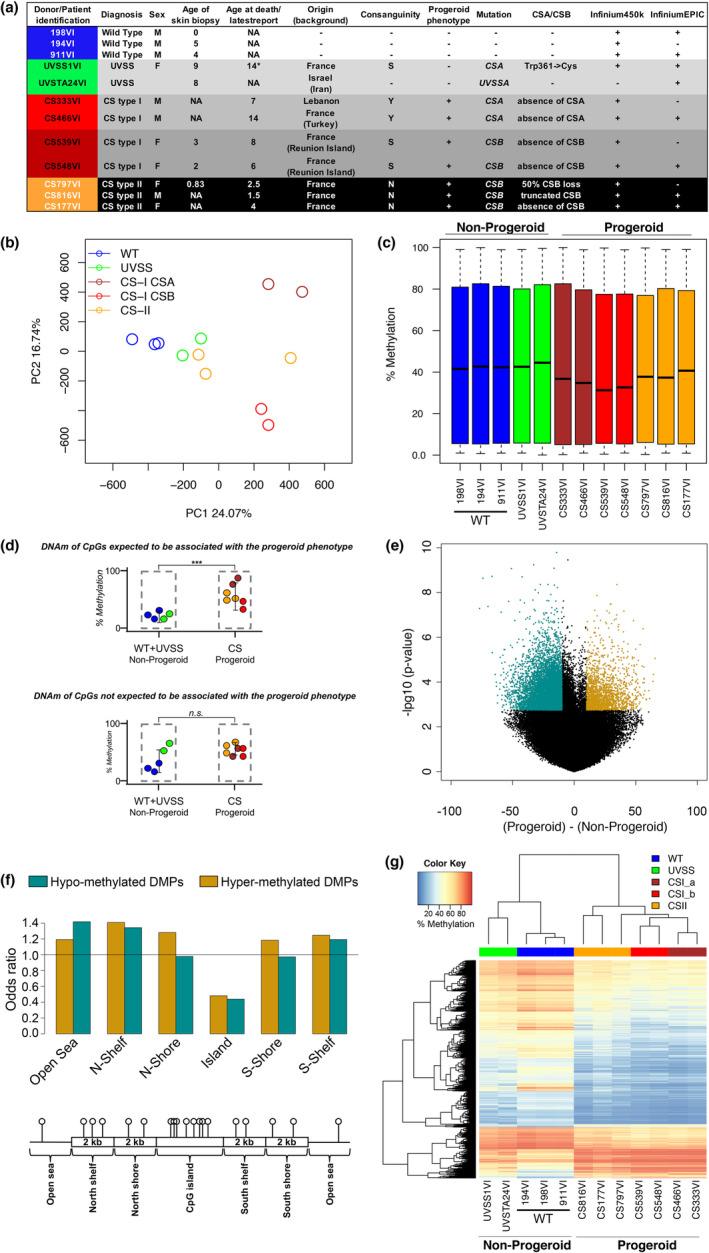
Global features of DNA methylation in CS skin fibroblasts and profile of the differentially methylated positions in progeroid versus non‐progeroid groups. (a) Characteristics of primary skin fibroblasts derived from three healthy donors (WT), two UVSS patients and seven CS patients. A plus indicates the platform used for the DNAm analysis (seven samples analysed with both platforms), and the presence of a progeroid phenotype in individuals. For each cell type is shown the age at skin biopsy, age at death/age at latest follow up. The star (*) indicates that the individual was alive at the indicated age (A. Sarasin, personal communication). All age columns are expressed in years. F, female; M, male; N, no; S, suspected; Y, yes. More information on these patients (except UVSTA24VI) is available in Laugel et al. (2010). (b) Principal component analysis (PCA) of DNAm levels of probes assessed in the Infinium450k and InfiniumEPIC beadchips (merged dataset), after batch correction; dots are coloured according to the pathological (CS, UVSS) or control (WT) condition. (c) Boxplot of global percentage of DNAm in the merged dataset after batch correction. (d) Graphical representation of the approach used to identify CS‐specific (progeroid‐related) epigenetic signature. ‘CS‐specific’ refers to epigenetic changes present in CS but not in UVSS samples that, although harbouring the mutation, do not show the progeroid phenotype. Progeroid‐associated epigenetic changes result from comparing DNAm between CS (‘Progeroid’ group) versus WT and UVSS cells, merged in the same ‘Non‐Progeroid’ group. A CS‐specific CpG site will tend to have similar DNAm values in CS samples and differ from those in WT and UVSS cells (top panel). On the contrary, a CpG site whose DNAm is associated with the phenotype shared between CS and UVSS, like photosensitivity, will have similar values in CS and UVSS cells and differ from WT cells (bottom panel). Analysis of variance between the progeroid and the non‐progeroid group will tend to return significant *p*‐values in the first but not in the second case. (e) Volcano plot showing nominal *p*‐values vs the difference in mean percentage of methylation between the progeroid and the non‐progeroid group. Green: hypomethylation; gold: hypermethylation. (f) Enrichment of DMPs in different genomic regions. Odds ratio values are reported on the y‐axis. Green: hypomethylation; gold: hypermethylation. The position of the various regions is schematized in the bottom. (g) Heatmap and hierarchical clustering on DMPs methylation values in the various patient and donor‐derived cells.

We analysed genome‐wide DNAm in dermal fibroblasts isolated from seven CS patients, two UVSS patients (including the only case mutated in *CSA*) and three healthy subjects (WT), with no siblings, and at similar and early passage number (PN 14). These fibroblasts are available because of single biopsies obtained for diagnostic purposes of the DNA repair defect, and are not repeatable longitudinally. An overview of the study design is reported in Figure S1. Samples were processed using the Infinium HumanMethylation450 BeadChip or Infinium MethylationEPIC BeadChip microarrays, as indicated in Figure 1a, and seven samples were assessed using both platforms. After data pre‐processing (see 5. Experimental procedures), we selected the probes that were in common between the two arrays (452,567 probes). In a first exploratory analysis, principal component analysis (PCA) was applied to all the probes of the microarray to identify the main sources of variability in genome‐wide DNA methylation profiles. PCA showed a separation of samples according to the type of platform on which they had been processed (Figure S2A); within each batch, samples tended to cluster according to the mutational state (Figure S2B). After applying the *ComBat* algorithm to adjust for the platform effect, no differences between the 450 k and EPIC samples were observed (Figure S2C), and samples still clustered together according to the mutational state (Figure S2D). Along the first component, mutated cells (UVSS and CS) were separated respect to WT cells, with the largest differences exhibited between WT and CS‐I cells (classical form). The second component identified a clear separation between CS‐I cells mutated for *CSA* and *CSB*, which, to date, are clinically indistinguishable (Laugel et al., 2010). However, CS‐I *CSA* and CS‐I *CSB* derived from male and female patients, respectively. We therefore removed the probes mapping on X and Y chromosomes (10,585 probes) and performed again the PCA analysis, which returned a result substantially overlapping the previous one (Figure S2E–H). Distinct CS‐I *CSA* and CS‐I *CSB* clusters were confirmed also upon removal of 1184 autosomic probes whose methylation level has been shown to be affected by sex in whole blood (Singmann et al., 2015; Figure S2I–L). The global pattern of methylation values was not affected by the ComBat batch correction (Figure S2M,N).

We performed the subsequent analyses using the batch‐corrected data, removing the probes on X and Y chromosomes (441,982 probes left) and, for the seven replicated samples, averaging methylation values from the 450 k and EPIC platforms. PCA and boxplots calculated on this merged dataset are reported in Figure 1b,c, respectively. Interestingly, boxplots of the methylation values showed a global hypomethylation in CS cells compared to WT and UVSS (Figure 1c).

PCA results reported in the previous paragraph suggested the existence of large genome‐wide DNA methylation differences between mutated and WT cells. Among the CpG sites showing DNAm changes, we aimed focusing on those specifically related to the progeroid phenotype that characterizes CS samples (independently from the subtype) and distinguishes them not only from WT, but also from UVSS samples, and thereby identify CS‐specific DNA methylation changes. For this, we compared CpG DNAm between CS‐derived fibroblasts (‘Progeroid’ group) versus WT and UVSS cells, merged in the same ‘Non‐Progeroid’ group, thus prioritizing CS‐specific epigenetic changes (progeroid‐related) and understating those associated with the impaired response to UV, common to CS and UVSS (Figure 1d).

We first searched for differentially methylated positions (DMPs) between the non‐progeroid and progeroid samples (Figure S1). Out of 441,982 analysed probes, we unveiled 11,597 DMPs (ANOVA; BH‐corrected *p‐*value < 0.05; absolute difference in mean DNAm values >10%; 2.6% of total probes), 9872 of which were hypomethylated and 1725 hypermethylated in the progeroid group (top‐ranking hits in Figure 1e and Tables S1, S2 columns A–X). About two third of DMPs (7277) mapped in a genic sequence. Results of the Fisher's exact test to assess whether DMPs are over‐ or under‐represented in specific genomic regions revealed that both hypo‐ and hypermethylated DMPs were under‐represented (*p*‐value < 0.05) in Islands (DNA segments of >200 bp in length, with high CpG density, >50% GC percentage, >60% observed/expected CpG ratio). Hypomethylated DMPs were strongly enriched in Open Sea (isolated CpGs in the genome) and Shelves (regions located within 2–4 kbp from CpG islands), whereas hypermethylated DMPs were enriched in Shores (regions located within 0–2 kbp from CpG islands), Shelves and Open Sea regions (Figure 1f). Unsupervised hierarchical clustering on the list of 11,597 DMPs confirmed a clear separation between the progeroid and non‐progeroid groups (Figure 1g). Furthermore, CS subtypes tended to cluster in separate subgroups, such as WT and UVSS cells.

To strengthen the analysis of genomic regions undergoing DNA methylation changes specifically in CS, we searched for differentially methylated regions (DMRs). We focused on probes located on CpG Islands and surrounding regions (Shores and Shelves) of gene‐associated regions, and applied a pipeline based on a Multivariate Analysis of Variance (MANOVA) of adjacent probes, which has been validated for relevant age‐associated changes (Bacalini et al., 2015). We identified 1817 DMRs in progeroid versus non‐progeroid groups, of which 1416 were hypomethylated and 401 hypermethylated (MANOVA BH‐corrected *p‐*value < 0.05; Table S3). The top‐ranking hits of DMRs list are reported in Table S4. These 1817 DMRs map in 1498 genes (1140 of which are hypomethylated and 358 hypermethylated), since some genes are covered by multiple DMRs.

Importantly, in several DMRs, UVSS samples displayed an intermediary DNAm profile between WT and CS samples (Figure S3A) rather than being close to WT (Figure S3B). We reasoned that some DNAm changes in this list could still be linked to impaired DNA repair (a feature common to UVSS and CS cells) rather than precocious ageing (only in CS). We thus filtered the DMRs list in order to retain only those that unambiguously separated progeroid (CS) samples and non‐progeroid (WT and UVSS) samples, thus having the highest probability of being implicated in the mechanism leading to precocious ageing (see Supplementary information, "Differential methylation analysis"). This approach resulted in a shorter and more stringent list of 222 hits that we named StringentDMRs (Figure S1 and Table S5), and contained 141 hypo‐ and 81 hypermethylated regions in progeroid compared to non‐progeroid. Hierarchical clustering on the StringentDMRs list confirmed a clear separation between the progeroid and non‐progeroid group, a clear clustering of CS subtypes, UVSS and WT cells in separate subgroups (Figure S3C), and the maintenance of top‐ranking position of individual genes compared to the non‐filtered DMRs list.

The forthy top‐ranking hits of this list are reported in Table 1. For each of these genes, a literature survey was done to identify those reported to play a functional role in ageing. Interestingly, about half (11/20) of the top hypomethylated genes have been functionally implicated in ageing or ageing‐related processes (e.g., senescence) by gain (overexpression) or loss (knock‐out/down) of function experiments in human cells or animal models (Table S6). Other genes have not been tested or have not shown a role (e.g., *ESYT3*) in ageing. Similarly, 43% (9/21) of the top hypermethylated genes have a functional role in ageing, some with a higher number of reports such as *RPTOR*, *HDAC4* and *ATP132A* (Table S6). The remaining top 12 hypermethylated genes have not been challenged in an ageing context.

**TABLE 1:** Top 20 hypo‐ and hypermethylated StringentDMRs of progeroid versus non‐progeroid conditions.

CHR	CpG island name	Relation to the CpG island	Gene(s)	Adjusted *p*‐value	Description
*Hypomethylated DMRs*					
**4**	**chr4:57521621–57522703**	**Island**	**HOPX**	**4.95E 04**	**dTF**
**19**	**chr19:12707696–12708114**	**Island**	**ZNF490**	**1.54E 03**	**TF**
3	chr3:138153269–138154621	N_Shore	ESYT3	**1.83E 03**	Transporter
17	chr17:18128577–18128821	Island	LLGL1	**1.90E 03**	Cytoskeleton/Axonogenesis
**2**	**chr2:71205563–71206529**	**N_Shore**	**ANKRD53**	**2.00E 03**	/
5	chr5:50683285–50683615	Island	ISL1	**2.75E 03**	dTF
3	chr3:138668635–138669323	Island	C3orf72	**2.91E 03**	/
1	chr1:1098043–1100584	S_Shelf	MIR429	**3.04E 03**	MicroRNA
1	chr1:34642382–34643024	Island	C1orf94	**3.04E 03**	/
6	chr6:31867691–31867957	N_Shore	EHMT2	**5.34E 03**	Methyltransferase
6	chr6:32975684–32975926	N_Shore	HLA DOA	**5.34E 03**	HLA clas II
5	chr5:1799461–1801905	Island	MRPL36/NDUFS6	**5.36E 03**	Mt Rb Mt complex/I
1	chr1:24742671–24743039	Island	NIPAL3	**5.41E 03**	Transporter
5	chr5:34915293–34916240	N_Shore	RAD1/BRIX1	**5.94E 03**	DNA repair/Rb biogenesis
**4**	**chr4:57521621–57522703**	**S_Shore**	**HOPX**	**6.10E 03**	**dTF**
5	chr5:50685453–50686148	N_Shore	ISL1	**6.34E 03**	dTF
9	chr9:140033235–140034176	Island	GRIN1	**6.40E 03**	Transporter
1	chr1:55266277–55267058	Island	TTC22	**6.59E 03**	/
14	chr14:23859356–23859620	N_Shore	MYH6/MIR208A	**6.95E 03**	Ms contraction/microRNA
**11**	**chr11:77790478–77791101**	**N_Shore**	**NDUFC2**	**8.45E 03**	**Mt complex I**

*Hypermethylated DMRs*					
**12**	**chr12:114838312–114838889**	**N_Shore**	**TBX5**	**1.54E 03**	**dTF**
**16**	**chr16:675906–676139**	**Island**	**RAB40C**	**1.83E 03**	/
**7**	**chr7:27154999–27155426**	**Island**	**HOXA3**	**4.20E 03**	**dTF**
**2**	**chr2:69614119–69614616**	**S_Shore**	**GFPT1**	**4.55E 03**	**Glucosamine**
**7**	**chr7:119915062–119915485**	**N_Shore**	**KCND2**	**4.94E 03**	**Transporter**
17	chr17:8126224–8126486	Island	C17orf44	**4.94E 03**	LINCRNA
**13**	**chr13:30088558–30088772**	**Island**	**SLC7A1**	**5.63E 03**	**Transporter**
8	chr8:72755783–72756667	S_Shore	MSC	**7.40E 03**	dTF
12	chr12:133102168–133102604	Island	FBRSL1	**1.22E 02**	/
**17**	**chr17:78880030–78880278**	**S_Shore**	**RPTOR**	**1.32E 02**	**mTOR regulation**
13	chr13:113536815–113537308	S_Shore	ATP11A	**1.39E 02**	Transporter
7	chr7:27219309–27219750	S_Shore	HOXA10	**1.45E 02**	dTF
6	chr6:43149736–43150009	N_Shore	CUL9	**1.47E 02**	Ubiquitin ligase
**5**	**chr5:169578–169798**	**N_Shore**	**PLEKHG4B**	**1.75E 02**	/
**2**	**chr2:240153476–240153792**	**N_Shore**	**HDAC4**	**1.76E 02**	**Deacetylase**
**6**	**chr6:10411394–10413857**	**Island**	**TFAP2A**	**2.01E 02**	**TF/dTF**
9	chr9:112081402–112082905	N_Shore	EPB41L4B	**2.01E 02**	/
1	chr1:17331885–17332093	Island	ATP13A2	**2.01E 02**	Transporter
22	chr22:19753312–19755013	Island	TBX1	**2.13E 02**	dTF
7	chr7:2774444–2774655	Island	GNA12	**2.17E 02**	Transducer
5	chr5:145214649–145215139	S_Shore	PRELID2	**2.17E‐02**	/

*Note*: DMRs are ranked according to the adjusted *p*‐value. Mapping information on DMRs are given by the chromosome number, the CpG Island coordinate, the relation respect to the CpG island and the associated gene. A description of each gene is also provided. Bold lines indicate StringentDMRs and corresponding genes assessed for expression.

Abbreviations: /, unknown; dTF, developmental transcription factor; Ms, muscle; Mt, mitochondrial; Rb, ribosome; TF, transcription factor.

### 2.2 Functional enrichment analysis of positions and regions with CS‐specific DNA methylation

To characterize the potential biological impact of CS‐specific DNAm changes, we performed a gene set enrichment analysis (GSEA) on the genes associated with methylated positions (MPs) and methylated regions (MRs) that resulted from the progeroid vs non‐progeroid comparison, using the Genome Ontology (GO) database. GSEA is a method that determines whether an a priori defined set of genes shows statistically significant concordant differences between two biological states (here progeroid versus non‐progeroid). Since both MPs and MRs results displayed high semantic redundancy of significantly enriched GO terms (i.e., Adjusted *p*‐value < 0.01), we removed the dispensable terms using REVIGO (Supek et al., 2011). The treemap visualization of the remaining terms (Biological processes), which allows hierarchical grouping of semantically related terms, identified ‘superclusters’ (meta‐terms), prevalently genes implicated in embryonic morphogenesis/development, and also transport of ions, synaptic transmission and cell–cell adhesion, that were shared among MPs (Figure S4A) and MRs (Figure S4B). This indicates that genes associated with MPs and MRs of CS, and thereby with the progeroid phenotype, are functionally comparable, and underscores the robustness of our analyses. To note, we observed an enrichment in neurotransmitters transport in MRs, and regulation of transcription in MPs.

**FIGURE 2:**
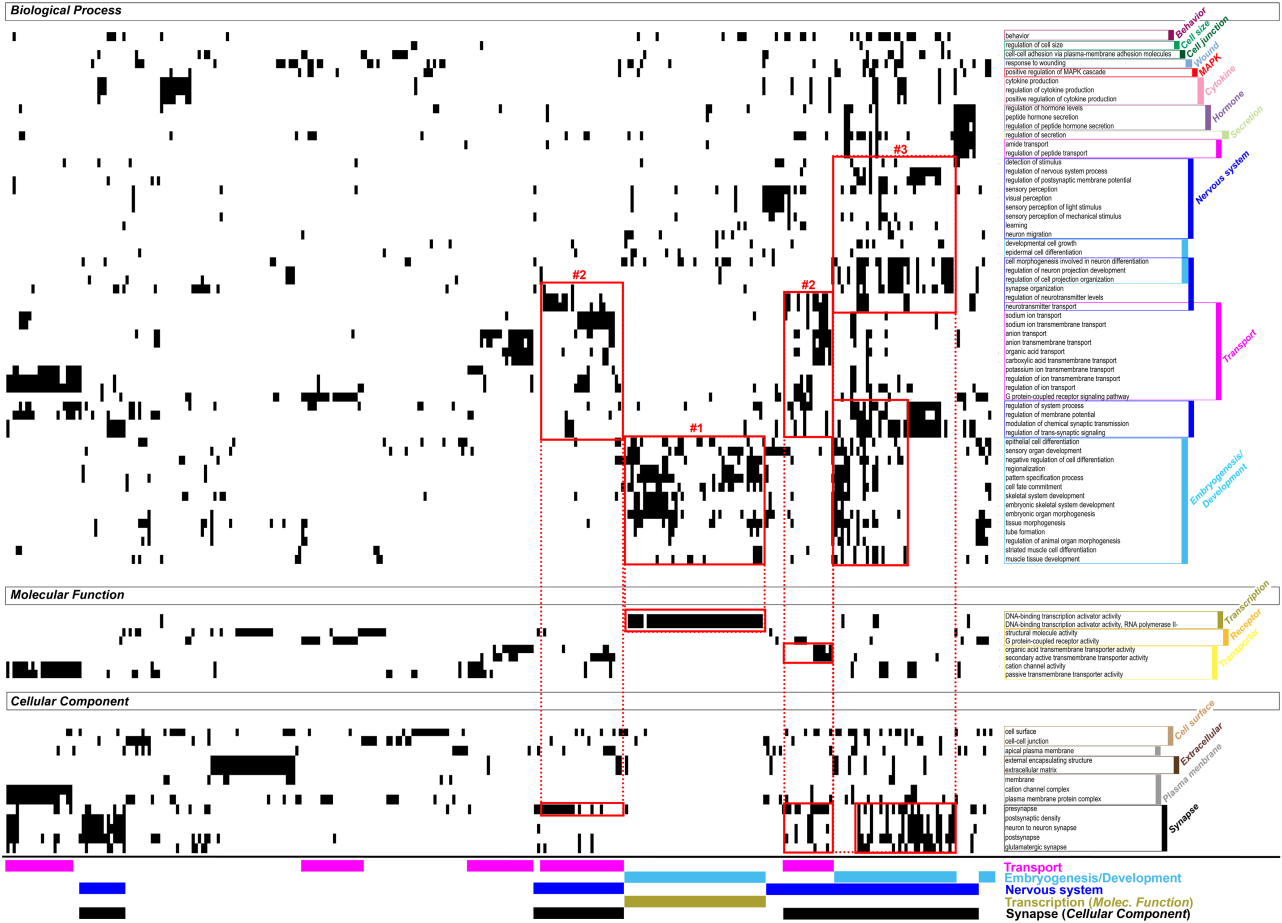
Filtering and functional GSEA of methylated regions in progeroid versus non‐progeroid groups. Plots reporting the correspondence between the enriched GO terms identified in the progeroid vs non‐progeroid GSEA analysis (right y‐axis) and the genes associated with DMRs, x‐axis. This representation displays genes in the x‐axis (listed in Table S7, sheet 3), and their corresponding GO term annotation on the y‐axis. GO terms on the y‐axis are grouped according to GO categories (Biological process, Molecular function, Cellular component). Genes may belong to more than one GO category as well as more than one GO term. Indeed, the same gene can be annotated with in multiple semantically similar terms, and the three GO categories provide complementary biological information. Numbered red frames indicate identified clusters of genes (related GO terms/GO categories); #1: transcription factors implicated in embryogenesis/development, #2: ion/neurotransmitter membrane transporters, #3: synaptic neuro‐developmental genes. These genes are more precisely identified because they belong to at least two independent GO categories (the colour code of the corresponding GO category/ies is/are reported below the x‐axis, and their name is reported on the right).

We then focused on the functional analysis related to MRs, that we assume to be more prone to influence the phenotype than MPs. We identified 80 GO terms significantly associated with methylation changes between progeroid and non‐progeroid samples (Figure 2). These GO terms include 59 Biological processes, 8 Molecular functions and 13 Cellular components that in total contained 6398 genes (Table S7, sheet 1), 544 of which were associated with DMRs also identified by our analysis (Figure S4C, listed in Table S7, sheet 2). We found that DMR‐associated genes are mainly enriched in these categories: activation of transcription, embryogenesis/development, nervous system/synapse and membrane transport (colour bars in the bottom of Figure 2, the individual genes are indicated in Table S7, sheet 3). Most of these GOs (64/80) have been associated with ageing‐related processes in other contexts (Table S7, sheet 3, columns LF‐LH).

To characterize the functional relevance of the 544 DMR‐associated genes, we simultaneously classified all genes according to their GO annotation, which was subdivided in the main GO categories (Biological process, Molecular function, Cellular component), with GO terms clustered by their semantic proximity (right side of Figure 2). This representation allows a more precise definition of differentially methylated genes in CS by revealing genes that belong to at least two independent GO categories (for instance the biological process ‘embryogenesis’ and the molecular function ‘transcription factor’). We thus observed that DMRs‐associated genes are principally enriched in transcription factors implicated in embryogenesis/development (red frame #1, Figure 2), ion/neurotransmitter membrane transporters (red frames #2) and synaptic neuro‐developmental genes (red frames #3). Genes present in only one of the three categories (Biological process or Molecular function or Cellular component) were reported separately (Figure S5). These last clusters included genes involved in skeletal and neuronal development, as well as cytokine production, sensory perception and hormone regulation (red frames #6, #5, #7, #8 and #4, respectively, Figure S5, the individual genes are indicated in Table S7, sheet 4), suggesting that they have a more tissue‐specific function than those reported in Figure 2.

We finally compared the 544 DMR‐associated genes of our GSEA analysis for their possible implication in human ageing. We used two resources, the AgingAtlas (Aging Atlas Consortium, 2021) and the GenAge (de Magalhaes et al., 2009) databases, which provide a short list of genes (502 and 307 genes, respectively) selected for their role in ageing. Despite the small size of these lists, we found a certain overlap with our 544 DMR‐associated genes (15 and 11 genes in common with AgingAtlas and GenAge, respectively; Table S7, sheet 2), six of which common to both lists.

### 2.3 Epigenetic hallmarks of accelerated ageing in CS and comparison with other progeroid syndromes and normal ageing

**FIGURE 3:**
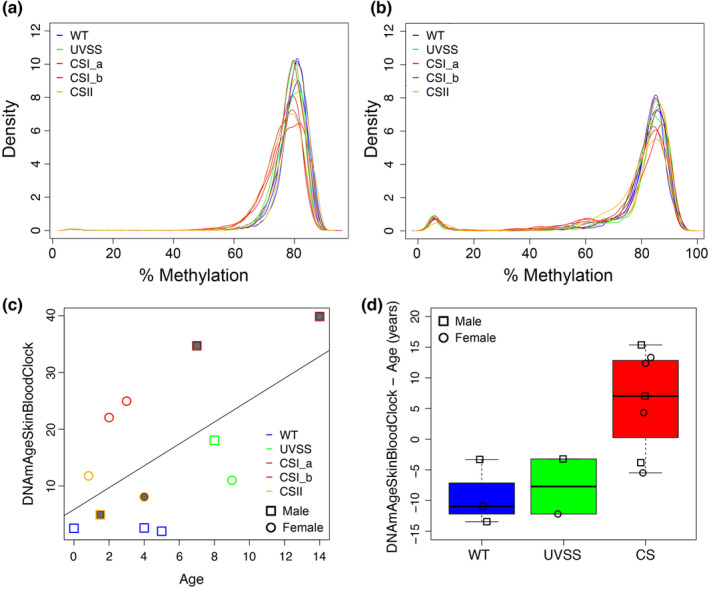
Epigenetic age acceleration in CS and commonalities with regular ageing. Density of DNAm of each sample for the repetitive Alu (a) and LINE‐1 (b) elements. (c) Scatter plots of epigenetic age, calculated with the Skin&Blood clock, vs chronological age. The black line represents the regression of epigenetic age on chronological age. The age of samples (only CS) with a filled symbol was calculated on the last report/death since the age at the time of biopsy was not known, thereby their chronological age (x‐axis) is likely overestimated. (d) Boxplots of epigenetic age acceleration values according to the Skin&Blood clock in WT, UVSS and CS groups.

To better characterize DNAm remodelling of progeroid CS, we evaluated possible hallmarks of epigenetic ageing in our dataset. First, we exploited the DNAm dataset to predict the methylation status of repetitive elements, which are consistently hypomethylated in ageing, using the *REMP* algorithm (Zheng et al., 2017). This analysis unveiled a clear hypomethylation of *Alu* sequences in CS samples, in particular in CS‐I, compared to WT and UVSS (Figure 3a). Conversely the DNAm pattern of LINE‐1 sequences did not appear to be correlated with the progeroid condition (Figure 3b).

We then evaluated our samples for their epigenetic age, which is considered a reliable marker of biological age (Horvath & Raj, 2018). Using the ‘Skin&Blood clock’, which was explicitly designed for in vitro studies with fibroblasts (Horvath & Raj, 2018), we found that CS samples tended to have an epigenetic age substantially higher than their chronological age (Figure 3c and Figure S6A). Epigenetic age acceleration, calculated as the residuals from the linear regression of epigenetic age on chronological age, was significantly higher in progeroid compared to the non‐progeroid (WT and UVSS) group (*p*‐value < 0.05; difference in mean residuals between the two groups: 15.5 years; Figure 3d and Figure S6B) and was particularly evident in CS‐I samples. Statistical significance was observed also upon correction for sex. Other available epigenetic clocks, like the pan‐tissue clock (Horvath, 2013; Figure S6E,F), the Hannum's clock (Hannum et al., 2013; Figure S6I,J), the PhenoAge (Levine et al., 2018; Figure S6M,N) and their derivates based on principal component analysis (Higgins‐Chen et al., 2022; Figure S6C,D,H,K,L,O,P), were not able to detect a significant epigenetic age acceleration in CS. This was in part expected, as these clocks were reported to perform poorly in fibroblasts (Horvath, 2013) or were developed for whole blood samples (Hannum's clock and PhenoAge). The analysis of an independent dataset including 31 neonatal and 7 adult untreated fibroblasts (GSE197724) confirmed that only the Skin&Blood was accurate in predicting the age of healthy control (WT) samples, supporting its higher reliability in these experimental settings (Turquoise symbols; all panels of Figure S6).

To assess whether CS‐specific DNAm changes that emerged from the progeroid vs non‐progeroid comparison are shared with normal ageing and/or other progeroid diseases, we assessed available Infinium datasets including: (1) other progeroid syndromes: HGPS (fibroblasts from the GSE196148 dataset), WS (whole blood; Guastafierro et al., 2017) and DS (whole blood; Horvath et al., 2015); and (2) mesenchymal stem cells (MSC) from individuals of different age, as a proxy of age‐associated changes in human fibroblasts (Fernandez et al., 2015). In HGPS, WS and DS datasets, we compared progeroid samples versus healthy controls; in the MSC dataset, we compared cells from old donors versus cells from young donors. For each dataset, we retrieved the lists of DMPs and DMRs, using the same analytical pipeline applied for CS dataset (Figure S1).

We first calculated the correlation matrix between DNAm changes observed in the four datasets (Materials and Methods). When we considered the entire set of microarray probes (452,567 probes; Figure S7A), the highest correlation was observed between WS and DS datasets (DNAm measured in whole blood in both the cases). CS dataset showed the highest correlations with MSC dataset (positive correlation) and with HGPS dataset (negative correlation). When we focused on the 11,597 CS‐DMPs (Table S2 and Figure S7B), the positive correlation of the CS‐MSC pair increased, suggesting that DNAm changes observed in CS partially resemble those related to normal ageing. On the other side, also the negative correlation of the CS‐HGPS increased, suggesting that DNAm is differentially remodelled in the two progeroid conditions.

These results were supported by an alternative analysis of the data, in which we evaluated the overlap between the DMPs and DMRs lists in the four comparisons (Table 2 and Figure S7C,D).

**TABLE 2:** Comparison of the CS‐specific DMPs/DMRs with those emerged in other progeroid conditions and normal ageing.

Comparison	In common with CS	Odd ratio	Hypomethylated/Hypermethylated	Concordant hypo−/hypermethylation (%)
Old vs Young MSC (DMPs)	1740	2.2	1252/488	70.40
HGPS vs CTR (DMPs)	1822	2.1	1062/760	51.21
WS vs CTR (DMPs)	1	–	0/1	0.006
DS vs CTR (DMPs)	118	1.85	44/74	39.83
Old vs Young MSC (DMRs)	605	2	421/184	62.15
HGPS vs CTR (DMRs)	455	2.1	244/211	44.17
WS vs CTR (DMRs)	0	–	–	–
DS vs CTR (DMRs)	302	1.5	104/198	40.73

*Note*: Number of common DMPs/DMRs between those identified in progeroid vs non‐progeroid (11,597 DMPs, 1817 DMRs) conditions and DMPs/DMRs identified in other studies performed in progeroid syndromes (HGPS, WS, and DS) or during normal ageing (MSC). For each, the odd ratios of Fisher's Exact Tests (*p*‐value < 0.05), the number (out of total) and percentage of concordant hyper‐/hypo‐DMPs/DMRs (same direction) is also provided.

As summarized in Table 2, out of 11,597 CS‐specific DMPs, 1822 (16%) were also detected in the HGPS dataset, 1 in the WS dataset, 118 (1%) in the DS dataset and 1740 (15%) in the MSC ageing dataset. Six DMPs were common to the CS, HGPS, DS and MSC datasets (Figure S7C and Table S2). With the exception of the WS‐DMPs, CS‐specific DMPs were significantly enriched in DMPs identified in the other experimental models (*p*‐value < 0.05, Fisher's exact test; odds ratio of 2.1, 1.85 and 2.2 for HGPS, DS and MSC datasets, respectively). More than 70% of the DMPs common to CS and MSC datasets showed the same direction of epigenetic changes (hyper‐ or hypomethylation compared to the respective controls) while this percentage was lower for HGPS and DS datasets (51.21 and 38.83% of concordant DMPs, respectively; Table 2 and Figure S7D,E).

We then confronted the 1817 CS‐specific DMRs with DMRs identified in the other datasets. No DMRs were identified in WS using the same significance threshold applied to the other datasets. Conversely, 25% (455 DMRs), 17% (302 DMRs) and 33% (605 DMRs) of CS‐specific DMRs were also significant in HGPS, DS and normal ageing, respectively (Table 2). These overlaps were greater than expected by chance (*p*‐value < 0.05, Fisher's exact test; odds ratio of 2.1, 1.5 and 2 for HGPS, DS and MSC datasets, respectively). Forty‐eight DMRs were common to all datasets (except WS; Figure S7F and Table S3, sheet 2). Differently from DMPs, the classification of DMRs as hyper‐ or hypo‐ methylated is not a straightforward task, as the same DMR can include both probes that gain and probes that lose methylation compared to controls. We assigned the DMRs as hypermethylated or hypomethylated by considering the methylation changes of the most significant CpG probe within each DMR, that is, the probe with the smallest BH‐corrected *p*‐value resulting from the ANOVA test. To note, also taking into account the complexity of defining the direction of methylation changes in DMRs, none of the 48 DMRs changed in the same direction in all categories (CS, HGPS, DS and normal ageing; Figure S7G,H). Similarly to what observed for DMPs, MSC ageing had the highest proportion of DMRs concordant with CS.

### 2.4 Correlation between CS‐specific DNAm and gene expression

**FIGURE 4:**
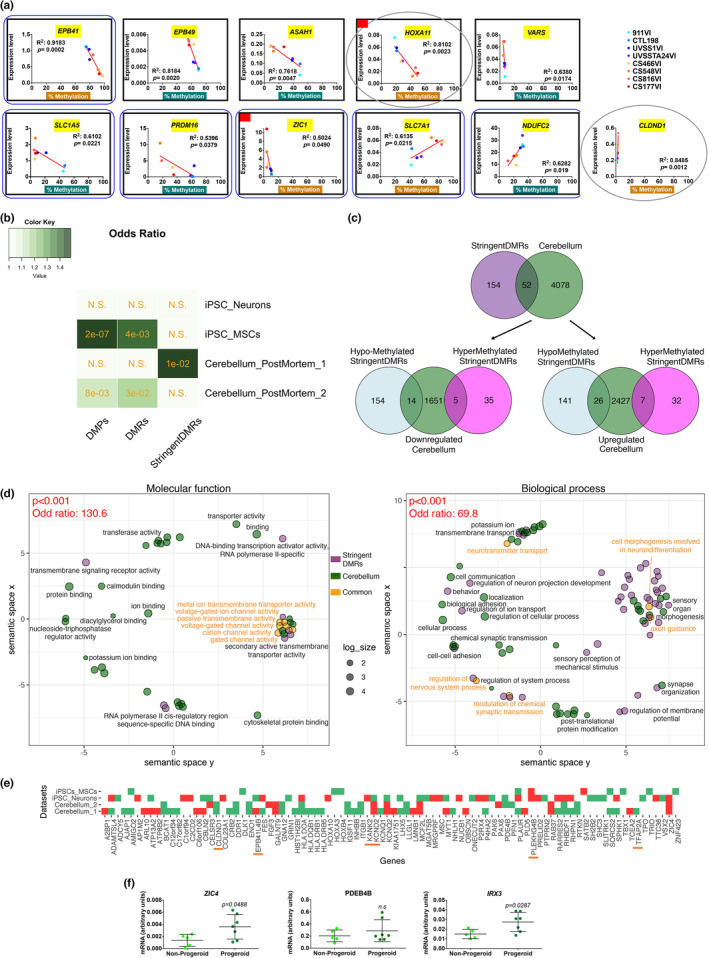
Correlation between DNA methylation and gene expression in skin fibroblasts and from different CS transcriptomic datasets. (a) For each cell type of our dataset, the expression of selected genes was plotted against the percentage of their DNAm. Out of 31 genes tested, 11 genes with a significant correlation between DNA methylation and gene expression are shown, eight of which were inversely correlated (*EPB41*, *EPB49*, *ASAH1*, *HOXA11*, *VARS*, *SLC1A5*, *PRDM16*, *ZIC1*), and three were directly correlated (*SLC7A1*, *NDUFC2*, *CLDND1*). The linear regression curve is indicated in red. The Pearson's correlation coefficient was used to assess the correlation between methylation and transcription levels. The R squared (R^2^) and *p‐value* (*p*) are indicated in each graph. The correlation was considered significant when *p* < 0.05 (gene name underscored in yellow). The characteristics of selected genes are indicated below. The direction of methylation changes (hypermethylation and hypomethylation in the progeroid vs non‐progeroid group) is indicated with gold and green highlight of the x‐axis label (% Methylation), respectively. Genes that showed differential methylation also in normal ageing and/or other progeroid diseases (10/11) are framed in black (rectangles). Genes present in the top list of DMRs and/or StringentDMRs (6/11) are framed in dark blue (rounded rectangles). Genes selected specifically for their function (*HOXA11*, *CLDND1*) are framed in grey (ovals). dTFs are identified with a red square on the left upper corner. *CLDND1* was selected for being specifically differentially methylated in CS. These characteristics/criteria are not mutually exclusive. The remaining 20 genes are shown in Figure S7. (b) Heatmap of odds ratios representing the strength of association between genes associated with DMPs, DMRs or StringentDMRs and genes differentially expressed in different CS transcriptomic datasets (from iPSC‐derived Neurons (Vessoni et al., 2016), iPSC‐derived MSCs (Wang et al., 2020) and *post‐mortem* Cerebellum [_1 (Wang et al., 2014) and _2 (Okur et al., 2020)]. The statistical significance of overlaps was tested with Fisher's exact tests, and the corresponding *p*‐values are indicated in orange. (c) Venn diagram showing the total number of common genes between genes associated with StringentDMRs and genes differentially expressed in Wang et al. (2014) (Post‐mortem Cerebellum_1) (*upper panel*). Venn diagrams displaying among the Hypo‐(blue) or Hypermethylated (magenta) StringentDMRs, number of genes in common with genes found Down‐ (*lower‐left panel*) or Up‐regulated (*lower‐right panel*) in the same dataset. (d) Clustering of semantically related GO terms (*left panel*; molecular function *right panel*; biological process) identified in GSEA (green circles) and in the enrichment analysis of Wang et al. (2014) (purple circles) using REVIGO. Common GO terms are represented by the orange circles. Semantically similar terms are grouped in clusters identified by a ‘meta‐term’ (black). The significativity of GO term overlap and odds ratios are displayed in red. (e) Heatmap representing the direction of expression changes (green; up‐regulated, red; down‐regulated) of the 88 unique genes in common between genes associated with StringentDMRs and genes differentially expressed in the four previously mentioned CS transcriptomic datasets. Genes already tested for expression in Figure 4a and Figure S7 are underlined in orange. (f) Quantitative RT‐qPCR of *ZIC4*, *PDE4B* and *IRX3* transcripts in the progeroid vs non‐progeroid groups. *ZIC4* is common to StringentDMRs and three out of four transcriptomic datasets (TDs); *PDE4B* is common to StringentDMRs and 2 out 4 CS TDs, and *IRX3* is common to DMRs and the 4 CS TDs. n = 3 independent experiments; mean ± SD; n.s, non‐significant; *p*, *p*‐value; unpaired *t‐test* vs non‐progeroid.

Transcriptomic data were not available from our samples; we therefore assessed by RT‐qPCR the expression of several key genes identified as differentially methylated in the progeroid vs non‐progeroid comparison. We selected DMRs according to one or more of the following criteria: the lowest *p‐*values, the lowest deltas of methylation between WT and UVSS, presence also in related datasets (normal ageing and/or other progeroid diseases, Figure 4a and Figure S8). Fourteen of the selected genes were part of the StringentDMRs list. The correlation between DNAm and transcription for each cell sample and for each of the 31 selected genes is shown in Figure 4a and Figure S8. Fifteen genes showed a strong (0.4 ≤ R2 ≤ 0.9) and seven genes a moderate (0.4 ≤ R2 ≤ 0.2) correlation between DNAm and RNA expression. The majority of tested genes displayed an inverse methylation/transcription correlation whereas 12 DMRs were poorly or not correlated with changes in gene expression. Importantly, significantly relevant inverse correlation was observed for eight genes (*EPB41*, *EPB49*, *ASAH1*, *HOXA11*, *VARS*, *SLC1A5*, *PRDM16*, *ZIC1*), and direct correlation for 3 genes (*SCLA7A1*, *NDFUC2*, *CLDND1*) that include genes common to multiple progeroid diseases and normal ageing.

We further assessed the correlation between on one side DMPs‐, DMRs‐ and StringentDMRs‐associated genes in our study and, on the other side, differentially expressed genes from multiple available CS transcriptomic datasets. These CS transcriptomes included induced pluripotent stem cells (iPSCs)‐derived neurons and mesenchymal stem cells, as well as two datasets from *post‐mortem* cerebella, and their respective healthy controls. For each dataset, Fisher's exact tests resulted in observed odds ratios greater than 1, revealing a global strong methylation/transcription association in CS (Figure 4b). Interestingly, the significance of the overlap progressively increased from the less to the more ageing‐specific list of differentially methylated genes (i.e., from DMPs to StringentsDMRs), and it was particularly strong for a transcriptomic dataset performed in *post‐mortem* human cerebella (Wang et al., 2014). Indeed, in this case 52 out of 206 genes associated with StringentDMRs (a few of the 222 StringentDMRs are located in the same gene) were differentially expressed in *post‐mortem* CS cerebella (Figure 4c), 60% of which displayed an inverse correlation (26 genes, i.e., half of the genes in this list, are hypomethylated/up‐regulated, and 5 genes are hypermethylated/down‐regulated). The other 40% of genes showed a direct correlation (14 genes: hypomethylated/down‐regulated; 7 genes: hypermethylated/up‐regulated; Figure 4c). Importantly, comparison of enrichment analyses demonstrated a strong association between the enriched GO terms identified [Odds ratio: 130.6 (Molecular function) and 69.8 (Biological process); Figure 4d] in the present DNA methylation study and in the transcriptome of CS cerebella from Wang et al. (2014). Moreover, in this last comparison, the semantic clustering of common enriched GO term with REVIGO revealed that genes displaying a methylation/transcription correlation are enriched in transmembrane ion transporters of the nervous system (Figure 4d).

Finally, out of the 206 StringentDMRs‐associated genes, 88 (43%) were differentially expressed in at least one transcriptome dataset (Figure 4e). Several StringentDMRs‐associated genes were similarly up‐ or down‐regulated in two different CS transcriptome datasets (e.g., *PDE4B*), and one, *ZIC4*, in three datasets (Figure 4e). Although no StringentDMRs‐associated gene was consistently differentially expressed in the four datasets, one gene associated with the DMR list, *IRX3* was identified in the four CS datasets. We then assessed the expression of these last three genes in the fibroblasts used in the present study. RT‐qPCR analysis revealed significantly higher levels of *ZIC4* and *IRX3* transcripts in CS (progeroid conditions) versus WT + UVSS cells (non‐progeroid conditions), and no changes for *PDE4B* (Figure 4f), indicating a possible correlation also in this paradigm.

## 3 DISCUSSION

The cause of premature ageing features in Cockayne syndrome remains largely unknown, and increasing evidences suggest that the DNA repair defect alone does not explain the progeroid phenotype (Velez‐Cruz & Egly, 2013). Our analysis relies on the methodological assumption that DNAm changes that are relevant for the progeroid phenotype of CS are not shared with UVSS (mutated but not progeroid). Using this approach, we identified a methylation signature that specifically characterizes CS and can be associated with its progeroid phenotype. Furthermore, using well established hallmarks of ageing, we confirmed the existence of an epigenetic basis of accelerated ageing in CS.

A constraint of this type of studies is that, due to the very low prevalence of CS and especially the UVSS conditions, this DNAm dataset has a limited sample size. This was also the case of recent studies on DNAm profiles in rare diseases that identified disease‐specific epigenetic signatures, and relied on a small number of samples (3–4 per group; Levy et al., 2022; Vento‐Tormo et al., 2016). Specific methods such as the *limma* R package have been developed to palliate the known limitation and power of differential expression analysis with few replicates, which we also used. Additionally, pointing to decrease the risk of false positives or biased associations inherent to the small sample size of the dataset, our methodological approach is based on the use of multiple and diverse statistical analyses of DNAm data, and the integration of complementary measurements (DNAm and expression), which collectively provide robustness to CS‐specific epigenomic signatures (Kolodziej‐Wojnar et al., 2020).

### 3.1 A CS‐specific epigenetic signature associated with the progeroid phenotype

To identify DNAm changes that are linked to the precocious ageing phenotype of CS patients, we compared ‘Progeroid’ (CS) vs ‘Non‐Progeroid’ (WT + UVSS) samples in a differential analysis. We identified 11,597 DMPs in CS, 85% of which were hypomethylated, consistent with a study performed during regular ageing in cultured cells (Fernandez et al., 2015) but differently from DMPs identified in progeroid WS and in the ageing blood, where only 45% and 46% of DMP were hypomethylated, respectively (Guastafierro et al., 2017; Marttila et al., 2015). Moreover, in CS, hypomethylated DMPs were enriched in isolated CpGs (Open Sea) whereas hypermethylated DMPs were enriched in regulatory regions flanking gene promoters (Shore, Shelf; Heyn et al., 2012; Madrigano et al., 2012). Thus, despite they represent the minority of DNAm changes, hypermethylated DMPs are more prone to impact on gene transcription (Ciccarone et al., 2018) in CS.

To better evaluate the impact of DNAm changes on genes, we focused on DMRs encompassing genic sequences. This analysis identified 1817 DMRs in CS, 78% of which were hypomethylated. These DMRs cover 1498 genes, since some DMRs are present more than once in the same gene. We finally applied a filtering to retain only the DMRs where CS samples clustered separately from WT + UVSS, and that are reasonably specific to the progeroid/neurodegenerative phenotype (uncoupled from the DNA repair deficiency). Using this pipeline and thanks to the pivotal role of UVSS samples (mutated but non‐progeroid), we identified a CS‐specific epigenetic signature highlighting a restricted list of 222 DMRs (64% hypomethylated). Successive steps of selection (from DMPs to DMRs, and then to StringentDMRs) obviously restricted the number of hits but, importantly, the main gene categories remained highly represented along the successive analyses (see below). Indeed, the top 20 hypo‐ and hypermethylated associated genes in the 222 StringentDMRs that constitute the CS epigenetic signature, in large part encode transcription factors implicated in developmental processes and membrane transporters (ions, amines, lipids) that is, two categories also found in DMRs analyses of single genes as well as pathways (see below). Importantly, the relevance of this concentric strategy to identify ageing‐related differentially methylated gene in CS was confirmed by the observation that half of the top hits correspond to genes functionally validated by experimental and genetic approaches in humans or animal models for playing a role in ageing or age‐related processes. Consequently, the other half of genes constitutes a pool of novel genes potentially involved in ageing or specific to accelerated ageing, revealed by our approach, and which will be necessary to validate in further studies.

We do not exclude that our analysis, in particular for StringentDMRs, has been too stringent and relevant genes have been excluded, and/or these findings could be complemented if novel available UVSS fibroblasts at a compatible passage number become available or additional significant CS cases are studied. However, we consider that the major common DNAm differences result from samples derived from patients with different genetic and clinical conditions.

Analysis of pathways rather than single genes, using a GSEA and the GO annotation, revealed GO terms that show statistically significant concordant differences between progeroid and non‐progeroid samples. Genes associated with DMRs were particularly enriched (high NES score) in the following biological processes/functions: (i) developmental transcription factors and regulators, (ii) ion/neurotransmitter transporters and (iii) synaptic neuro‐developmental genes, in remarkable agreement with single gene analyses above.

i. Gene enrichment in embryogenesis/developmental transcription factors (dTFs) prevails over all other GO terms, suggesting a major implication in CS‐specific defects, and to a larger extent than observed in other precocious ageing diseases (Horvath et al., 2015) and during regular ageing (Marttila et al., 2015). Several dTFs, which are differentially methylated in CS, have been linked to ageing‐related processes, as the homeobox gene *HOXA9* (Schworer et al., 2016 *)*, TFs of the T‐box (*TBX1*, *4*, *5*, *15*) and Fox (*FOXA1*, *FOXF1*) families, Wnt transcription regulators (*WNT7A*, *WNT8B*, *FDZ1*, *FDZ2*, *FDZ7*), and GATA, SOX, and PAX family members (Foronda et al., 2014; Stanescu et al., 2007; Zimmermann et al., 2019). These examples further underscore the relevance of the CS model for ageing. To note, a fraction of these genes, including HAND (*HAND1*, *2*), LHX (*LHX2*, *3*), NKX (*NKX2‐5*, *2–8*, *6–1*) and PITX (*PITX1*, *2*) families, to our knowledge have not been associated with ageing, possibly being novel developmental regulators factors that play a role in CS as well as in regular ageing. *HOXA11*, *ZIC1*, and to some extent *HOXA9* demonstrated a strong correlation between DNAm and expression levels in patient fibroblasts, suggesting a functional impact of these CS‐specific methylations changes.

These findings are consistent with the quasi‐programmed theory of ageing that claims detrimental over‐activity of developmental factors during ageing (Blagosklonny, 2013). According to this theory, if processes important early in life are not properly switched off in the adult, they progressively lead to ageing‐related alterations. Accordingly, developmental genes are normally tightly controlled and fail to be so during ageing (Yang et al., 2018). We report modifications of DNAm in CS that are possibly responsible for deregulation of developmental genes, suggesting that this mechanism, whether or not of stochastic origin, plays a role in the ageing phenotype.

Consistently, one of the main ‘rejuvenating’ interventions proposed to delay ageing is partial cellular reprogramming (Ocampo et al., 2016), which, by transiently turning on master regulators (*OCT4*, *KLF4*, *SOX2*, c*MYC*) involves re‐activation of developmental programmes (Wang et al., 2020). Interestingly, the amelioration of ageing signs during partial reprogramming was shown to be driven by epigenomic remodelling (Ocampo et al., 2016), which supports the notion of a link between DNAm changes and developmental TFs unveiled in our study. Indeed, partial reprogramming was described to lead to a reduction of epigenetic age in human cells (DNAm‐based clock; Olova et al., 2019), and restoration of youthful whole DNA methylation patterns in old mice (Lu et al., 2020).

ii. Functional analysis of CS methylated regions reveals enrichment also in ion/neurotransmitter transporters, both passive and active carriers. Passive transporters were mainly voltage‐gated potassium channels (*KCNA3*, *KCNA4*, *KCNC2*, *KCND2*, *KCNE4*, *KCNG4*, *KCNH1*, *KCNH2*, *KCNIP4*, *KCNQ1*), which are implicated in regulating the membrane potential in excitable cells like neuronal or muscle cells (Zironi et al., 2010), but are also expressed in fibroblasts (Li et al., 2009). The involvement of these transporters in neurodegeneration has been evoked in several neurodegenerative diseases (i.e., Alzheimer's and Parkinson's disease; Sesti, 2016), and they may play a role in neurodegeneration in CS. Interestingly, remodelling of K^+^ channels in dermal fibroblasts has been associated with the successful ageing of centenarians (Zironi et al., 2010), whereas overexpression of K^+^ channels was observed in HGPS cells (Zironi et al., 2018).

Genes for active transmembrane transporters mainly involve the solute carrier group of transport proteins (SLCs) that carry a wide variety of substrates including ions, nutrients and/or neurotransmitters through plasma/organelle membranes, also in fibroblasts (De Sanctis et al., 2016). Only a few members of this large family of transporters (>400 proteins) have been associated with ageing (Chekroud et al., 2012). *SLC1A5* and *SLC7A1* showed a strong direct or inverse correlation, respectively, with DNAm levels in CS patient and control samples.

iii. Gene enrichment in the functional analysis of CS methylated regions also identified synaptic neuro‐developmental genes. This finding is of relevance since progeroid CS is also characterized by brain and nervous system abnormalities (Karikkineth et al., 2017), providing a novel pool of targets to analyse in the context of age‐related neuronal pathophysiology (e.g., neurodegeneration).

To note, additional genes with just one of these characteristics, namely developmental genes, membrane transporters and synapsis‐related genes, were also enriched in our analysis. Further, we observed components of the cell surface and extracellular compartment, including different types of cell adhesion, namely adherens junctions (*ACTN1*, *APC*, *CDH3*, *4*, *7*, *15*, *JUP*, *DSP*), tight junctions (*CLDN6*, *10*) and gap junctions (*GJA4*, *GJA8*, *GJC1*). The expression of *CLDN1* strongly correlated with DNAm levels in our samples. Cell adhesion factors play an important role in maintaining tissue architecture as well as tissue homeostasis due to their involvement in the formation of epithelial barriers that regulate exchanges, and is therefore not surprising that they are possibly involved in ageing (Parrish, 2017). Accordingly, higher expression of protein involved in intercellular (tight) junctions was recently found in healthy centenarians compared to controls (Santos‐Lozano et al., 2020). The role of these junctions, particularly during ageing, remains poorly understood outside epithelia to which they seem restricted, despite fibroblasts express adhesion molecules (Ko et al., 2000).

### 3.2 Epigenetic hallmarks of accelerated ageing in CS

We applied to our dataset a methylation age predictor specifically developed for human fibroblasts (Horvath & Raj, 2018), which outperforms the pan‐tissue age estimator (Horvath, 2013) and other clocks for this cell type. Using the Skin&Blood clock, we showed acceleration of epigenetic age specifically in CS, but not in UVSS. Although we cannot exclude that this observation is related to the small sample size and/or uneven distribution of sexes across the groups, we think that these possibilities are unlikely. Indeed, epigenetic age calculated using the Skin&Blood clock was highly correlated with chronological age in an independent dataset, and these additional WT samples showed age acceleration values similar to WT and UVSS samples in our study. Moreover, whereas the pan‐tissue epigenetic clock has been shown to return higher epigenetic ages in males compared to females, this has not been reported for the Skin&Blood clock; accordingly, epigenetic age acceleration was significantly larger in the progeroid compared to the non‐progeroid group also when correcting for sex as possible confounding factor. Notably, the mean epigenetic age acceleration in CS was at least 15.5 years, that is, higher than those previously reported in blood from WS or DS (addition of 6.4 and 6.6 years, respectively, for 10–60 year old patients) (Horvath et al., 2015; Maierhofer et al., 2017), suggesting that CS patients display an extremely accelerated ageing. To note, in several CS cases we have likely underestimated the gap between chronological and epigenetic age, as the age of patients at the time of biopsy was not reported, and we calculated instead the date of the latest report and/or death. The present data are in agreement with the accelerated ageing that we previously reported in six patients with CS [aged 3, 7, 8, 10 (twice) and 20 years] using a totally different test, that is, the GlycoAgeTest, that measures the age‐associated shift in serum N‐glycan profile (Vanhooren et al., 2010). The fact that two distinct biomarkers of age (the epigenetic clock and the GlycoAgeTest) concordantly showed accelerated ageing in CS patients is remarkable, and mirrors what previously observed in DS, although at a much larger extent (Borelli et al., 2015; Horvath et al., 2015).

In addition, CS fibroblasts displayed other epigenetic changes that have been largely reported in studies on ageing (Ciccarone et al., 2018). CS cells tended to have lower DNAm values compared to UVSS or WT cells, resembling the global hypomethylation described in physiological ageing contexts (Madrigano et al., 2012), and ascribed to heterochromatin loss in non‐genic repetitive sequences with ageing (Ciccarone et al., 2018). By inferring the methylation of locus‐specific repetitive elements from microarray data, we found that CS cells displayed a marked hypomethylation of *Alu* elements, while LINE elements were not affected by the syndrome. Thus, the methylation patterns of CS cells exacerbate those that are characteristic of normal ageing (Cardelli, 2018). This result seems specific for our dataset, as in whole blood from WS patients no differences in DNAm of repetitive elements (experimentally measured) were found compared to healthy controls (Maierhofer et al., 2019), or this is due to differences between fibroblasts and blood cells.

### 3.3 CS has DNAm characteristics of regular ageing and common marks with progeroid conditions

The CS‐specific epigenetic signature was compared with physiological ageing (Fernandez et al., 2015) and other progeroid diseases [DS (Horvath et al., 2015), WS (Guastafierro et al., 2017), HGPS (GSE196148 data from GL)]. Each progeroid disease is characterized by distinct features. For instance, HGPS patients do not display neurodegeneration whereas CS patients do, and include neuronal loss, demyelination, gliosis and calcification, that unevenly affect different parts of the CS brain (Karikkineth et al., 2017). As another example, WS patients display an increased propensity to develop cancer, whereas HGPS and CS patients typically do not. Other clinical symptoms as growth impairment and skin abnormalities are shared among progeroid diseases and may result from common defective pathways.

Collectively, data from previous studies (Heyn et al., 2013; Horvath et al., 2015; Kohler et al., 2020; Maierhofer et al., 2019) and our new analyses demonstrate that epigenetic remodelling is a common hallmark of progeroid syndromes. At the same time, as pointed out in the seminal study of Martin (1978), commonalities and differences exist for changes occurring in progeroid disorders versus physiological ageing. Our study contributes disentangling and clarifying the epigenetic basis of this insight. Our data suggest that CS DNAm changes are closer to normal ageing than other progeroid diseases. Indeed, CS had 15% of DMPs in common with normal ageing, whereas WS whole blood cells only 6% (Maierhofer et al., 2019), and epigenetic changes in HGPS fibroblasts differ from those occurring during physiological ageing (Kohler et al., 2020) confirming CS as a precocious ageing disorder. Importantly, the DNAm datasets that we analysed differ in size and derive from different tissues (fibroblasts for CS and HGPS, MSCs for normal ageing, whole blood for DS and WS), a factor that may affect our results.

Remarkably, several CS characteristics were distinct from normal ageing. For instance, significant hypomethylated regions were more numerous in CS than normal ageing, which rather displayed hypermethylated regions (Marttila et al., 2015). Moreover, in normal ageing hypermethylation was associated with developmental processes and DNA binding/transcription, whereas hypomethylation did not enrich a specific set of genes. Genes involved in transcriptional regulation of developmental processes, as indicated above, were prevalently hypomethylated in CS. Similarly, the enriched functional GO categories identified by our GSEA analysis are prevalently composed (>95%) of genes unknown in databases of human age‐related genes, but these databases are far from being exhaustive.

From the CS‐specific pool, we identified 48 DMR‐related genes that are common to the other datasets analysed (normal ageing, HGPS, DS). Among them, the expression of *EPB49*, *KANK2*, and to some extent also *HOXA9*, was inversely correlated with DNAm, suggesting that these methylation changes are relevant for CS. *EPB49* has been recently associated, together with other components of the haem metabolism, with human ageing (Timmers et al., 2020). Other genes in this list, like *NAGS*, coding for a N‐acetyl glutamate synthase linked to glutamine/glutamate metabolism, and *GNL1*, coding for a little‐known GTPase reported to modulate cell proliferation (Krishnan et al., 2018), have not been associated with ageing, and the function of the protein coded by *FAM43A* is still unknown.

### 3.4 Top‐ranked genes with DNAm changes display a good correlation with expression changes

A general trend in ageing‐related DNAm studies is that changes (hypo‐ or hypermethylation) are not necessarily correlated with changes in gene expression, even in regions with a highly reproducible epigenetic remodelling during ageing that define the ‘methylation clock’ (Horvath, 2013). We first analysed transcription levels of key genes directly in patient‐derived fibroblasts. Transcription of 31 genes that displayed the highest DNAm changes in CS (top 50 hypo/hypermethylated genes) and/or were in common with other ageing datasets, unveiled a tendency to inverse correlation (high expression vs low methylation levels), although direct correlation was observed too, and one third of cases was statistically robust. The remaining cases showed no correlation or a tendency to direct correlation, at least within the limited number of patient samples available. The DNAm/transcription correlation appears remarkably larger than in reported cases of ageing‐related datasets (for instance less than 4% in normal ageing; Marttila et al., 2015), perhaps as a result of the robustness of our data analysis (DMR instead of DMP, multiple filtering processes based on biological assumptions), or a characteristic of DNAm changes in CS. Moreover, we may have underestimated the correlation between DNAm and transcription, since our experimental procedures (i.e., the use of Infinium microarray on bisulfite‐treated DNA), like the published databases that we used for comparisons, do not distinguish DNA methylation from DNA hydroxymethylation. The latter epigenetic modification reported a more direct correlation with transcription in ageing fibroblasts.

To further understand the consequence of DNAm changes on gene expression, as genome‐wide transcriptomic data are not available in primary fibroblasts used in this study, DNAm/transcription correlations were assessed using four published CS transcriptomes performed in *post‐mortem* cerebella (Okur et al., 2020; Wang et al., 2014), iPSC‐derived neurons (Vessoni et al., 2016) and MSCs (Wang et al., 2020). These datasets were relevant because they were originated from patient‐derived cells or native organs, allowing a comparison with patients‐derived fibroblasts in our study, and in the absence of immortalization processes, which may interfere with pathways implicated in ageing. All these datasets displayed a strong association (Odd ratios > 1) with DNAm changes observed in our study (at the level of DMPs and DMRs, or StringentDMRs). It should be underscored that expression changes observed in these datasets may likely include changes occurring also in UVSS cells, and that are consequently linked to DNA repair deficiency rather than the precocious ageing defects. To reduce this possible bias, we focused on the CS transcriptome of cerebella from Wang et al. (2014) that displayed the strongest and most significant association with expression changes observed in StringentDMRs, where DNAm changes in common with UVSS have been removed. Results confirmed a robust DNAm/expression correlation, obviously at a larger scale than the one assessed in patient fibroblasts where only 31 top‐ranked genes were tested. To note, this large‐scale correlation was strong despite DNAm data originated from CS patient‐derived fibroblasts in culture, and transcriptome data from *post‐mortem* cerebella of other CS patients (i.e., carrying distinct clinical defects and diverse genomic backgrounds), underscoring the robustness of our findings. We are aware that other DNAm changes may not be associated with transcription alterations, and further genome‐wide studies will determine case by case when this occurs. Interestingly, this analysis identified expression changes mainly in genes enriched in transmembrane ion channels of the nervous system, in agreement with neurodegeneration observed in CS patients and also during regular ageing (Sesti, 2016). Among the genes identified in this analysis, the differentially methylated genes *ZIC4* and *IRX3* were of particular interest as they were consistently down‐regulated or up‐regulated, respectively, in multiple CS transcriptomic datasets. In patient‐derived fibroblasts tested here, both *ZIC4* and *IRX3* were up‐regulated. Consistent upregulation of *IRX3* in most datasets from different patients, and distinct cells/native organs makes it an interesting candidate for future mechanistic studies.

## 4 CONCLUSIONS

In summary, we identified CS‐specific DNAm changes by filtering out changes not associated with the progeroid and neurodegenerative phenotype, a unique characteristic of this pathological paradigm. Functional analyses revealed enrichment in developmental transcription factors, ion/neurotransmitter transporters and synaptic neuro‐developmental genes, that appear thus critical for accelerated ageing/neurodegeneration. A large fraction of DNAm changes have been associated with expression changes in available datasets, in particular *post‐mortem* cerebella. In addition to epigenetic signatures in common between CS and physiological ageing, DNAm changes observed in CS may help identifying transcriptional changes that would appear at a later time in regular ageing. Finally, our results demonstrate that CS is a pertinent model to study ageing and unveil pathways and genes that could be relevant for future mechanistic studies.

## 5 EXPERIMENTAL PROCEDURES

### 5.1 Patient cells

Patient fibroblasts were derived from skin biopsies excised from unexposed body sites by dermatologists. Patients (or parents or legal guardians) provided informed consent to receive diagnosis and genetic analysis. The French Agency of Biomedicine (Paris, France) (Arrêté n°2001/904 and Ref: AG08‐0321 GEN of 27/09/2008; http://www.agence‐biomedicine.fr/Genetique) and the European Commission ‘Geneskin: Genetics of human genodermatosis’ (Brussels, Belgium, 2008) approved this study. UVSTA24VI (also called UVs24TA) were a kind gift of Dr. Graciela Spivak, Stanford University, USA. HGPS fibroblasts were from the BioLaM biobank at IGM‐CNR, Bologna, Italy (IRCCS Istituto Ortopedico Rizzoli Ethical Committee approval no. 0018250–2016).

### 5.2 Differential methylation analysis

To identify DNA methylation differences between progeroid and non‐progeroid groups, probes on X and Y chromosomes were removed. Analysis of variance (ANOVA), as well as the application of a 10% delta methylation cut‐off, was used to identify differentially methylated positions (DMPs). To identify differentially methylated regions (DMRs), we applied multivariate analysis of variance (MANOVA) on sliding windows of three chromosomally adjacent probes, as described in Bacalini et al. (2015). More details are in the Supplementary information. In order to verify the robustness of our finding, we compared this pipeline with other analytical approaches. The use of the *limma* R package, that was developed to power differential expression analysis with few replicates (Ritchie et al., 2015) and has been successfully applied to expression and DNAm datasets with small sample sizes (Levy et al., 2022; Vento‐Tormo et al., 2016), resulted in an almost identical list of DMPs (98% of overlap; Table S2). We also used DMRcate as an alternative method to identify DMRs. 930 out 1817 DMRs selected by our method were found also by DMRcate, and 542 of them ranked among the top 1000 DMRs identified by our approach (Table S3, sheet 1, column P). Importantly, out of 222 stringent DMRs that we found by our analytical pipeline, 191 were also identified using DMRcate (Table S3).

### 5.3 Functional enrichment analysis

The functional scoring of the genes associated with MPs and MRs was done using Gene Set Enrichment Analysis (GSEA) as implemented in *MethylGSA* (Subramanian et al., 2005).

### 5.4 Comparison with other methylation datasets

Publicly available Infinium450k (GSE52114, GSE52588; and GSE196148, this study) and InfiniumEPIC (GSE100825) datasets were downloaded from the GEO database. GSE52114 includes DNAm profiling of mesenchymal stem cells obtained from 22 young individuals (age range 2–29 years) and 12 old individuals (age range 61–92 years). GSE52588 includes DNAm profiling of whole blood DNA from 29 subjects affected by DS (age range 10–43 years), their unaffected siblings (age range 9–52 years) and their mothers (age range 41–83 years). GSE196148 includes DNAm profiling of fibroblasts from one HGPS patient (6 years; three replicates) and two healthy controls (12 and 15 years, each assessed in two replicates). GSE100825 includes DNAm profiling of three subjects affected by WS (age range 44–53 years) and three age‐ and sex‐ matched healthy controls. In each dataset, DNAm differences between progeroid patients and healthy controls were assessed using the same analytical pipeline (DMPs using the threshold of 10% for DNAm difference, and DMRs) used for CS samples. The *corrplot* R package was used to calculate the correlation matrix between DNAm changes in the four datasets. DNAm changes were calculated as the difference between mean DNAm values in diseased samples and DNAm values in controls (for CS, HGPS and DS datasets), or as the difference between mean DNAm values in old samples and DNAm values in young samples (for MSC dataset).

### 5.5 Correlation with CS transcriptomic datasets

When available (Vessoni et al., 2016; Wang et al., 2014), the list of significant differentially expressed genes in CS cells (vs WT cells; cut‐off adjusted *p*‐value < 0.05 and fold change > 1) where directly extracted from publications. Otherwise, datasets were downloaded from the GEO database. Differential expression analyses were performed on the GSE144557 dataset (Okur et al., 2020) using the GEO2R tool available on the NCBI website, and on the GSE124208 dataset (Wang et al., 2020) using the *DESeq2* Bioconductor package (Love et al., 2014). The list of CS enriched GO terms from Wang et al. (2014) was extracted from publication and submitted to REVIGO (see Supplementary information) to remove redundant terms and organize remaining ones in clusters of semantic proximity. Enrichment analyses to assess gene overlap were performed using Fisher's exact test implemented in the R *GeneOverlap* package. Venn diagrams, scatter plots from REVIGO and Heatmap were generated using the *ggplot2* R package.

Additional experimental procedures are in Supplementary information.

## AUTHOR CONTRIBUTIONS

MGB, CCh and CCr performed biostatistical analyses (MGB: Differential methylation analysis and comparison with other datasets; CCh and CCr: Functional analysis), analysed results and wrote the manuscript. CCr performed bench experiments and supervised the genome‐wide DNAm experiments. PG and CF provided advice for the biostatistics analysis and, together with AS discussed results and contributed to the manuscript. GL provided unpublished data on DNAm of HGPS fibroblasts, and SH provided the unpublished methylation clock for fibroblasts; both contributed to the manuscript. MR planned the experiments together with CCr, analysed results and wrote the manuscript.

## FUNDING INFORMATION

This work was supported by Agence Nationale de la Recherche (CS_AGE, aapg2019), Institut Pasteur DARRI (DISAGE, PasteurInnov2014) and Institut Pasteur PTR (PTR111‐2017).

## CONFLICT OF INTEREST STATEMENT

The authors declare that they have no conflict of interests.

## Supporting information


Data S1:

## Data Availability

Raw and processed DNAm data are available at the Gene Expression Omnibus (GEO) database under the accession number GSE163841. All other data generated or analysed in this study are included in this manuscript (and its supporting information files).
