# Supplementary material for: Epigenomic signature of accelerated ageing in progeroid Cockayne syndrome
Source: Aging Cell. 2023 Sep 8;22(10):e13959. doi: 10.1111/acel.13959 (PMC10577576; doi:10.1111/acel.13959)
Supplement: Supplementary file 1 — Data S1: [file ACEL-22-e13959-s001.zip › acel13959-sup-0001-Supinfo/acel13959-sup-0001-Supinfo/Supplementary Information_Revision_ Crochemore et al.pdf]

Supplementary Information for

Epigenomic signature of accelerated ageing in progeroid Cockayne syndrome

Clément Crochemore, Claudia Chica, Paolo Garagnani, Giovanna Lattanzi, Steve Horvath, Alain Sarasin, Claudio Franceschi, Maria Giulia Bacalini†, and Miria Ricchetti†

†co-last authors

Corresponding authors: Miria Ricchetti and Maria Giulia Bacalini
Email: miria.ricchetti@pasteur.fr and mariagiuli.bacalini2@unibo.it

This PDF file includes:

Supplementary Experimental information
Figures S1 to S8
Table S1, S4, and S6
Supplementary References

Other supplementary materials for this manuscript include the following:

Table S2, Table S3, Table S5, and Table S7

Supplementary Experimental procedures

Cell culture conditions

Healthy, UVSS and CS cells (detailed in Fig. 1A) initially thawed at passage number (PN) 12 were cultured in Dulbecco's modified eagle medium (DMEM; Gibco) supplemented with 2mM L-glutamine (GlutaMAX™; Gibco), 10% foetal bovine serum (FBS; Gibco), 1% Penicillin-streptomycin (Gibco) in 20% O_2/5% CO_2 at 37 °C. Cells were harvested at PN14 for further experiments. All cells were cultured and harvested in the same conditions.

DNA extraction, bisulfite treatment and genome-wide DNA methylation analysis

Total genomic DNA (gDNA) from WT, UVSS and CS primary skin fibroblasts was isolated using the QIamp® DNA mini kit (Cat#51304; Qiagen) and used for microarray-based analysis of genome wide DNA methylation levels in two different sets (see Fig. 1A for detail of cells used for each set). For the first set, extracted gDNA was quantified by NanoDrop (ND-1000; Thermo Fisher Scientific) and 1µg was bisulfite-treated using the EZ DNA Methylation™ kit (Cat#D5001; Zymo Research), according to manufacturer instructions. Genome-wide DNA methylation analysis was performed on this material by Aros Applied Biotechnology A/S (Eurofins Genomics; Denmark) using the Infinium HumanMethylation450 BeadChip (Illumina). For the second set, extracted gDNA was directly send to Aros Applied Biotechnology A/S for bisulfite conversion and genome-wide DNA methylation using the Infinium MethylationEPIC BeadChip (Illumina) following manufacturer's instructions. The Infinium Methylation BeadChips allows quantitative investigation of methylation of hundreds of thousands of CpG sites widespread across the genome, comprising sites within and outside CpG islands, mapping both in genes (including also microRNAs) and in regions not associated with genes.

The *minfi* Bioconductor package was used to extract raw signal intensities from each experimental set (Infinium HumanMethylation450 and Infinium MethylationEPIC BeadChips) (1). All the samples were retained after preliminary quality checks (less than 1% of probes with a detection pvalue >0.05). The preprocessNoob function implemented in *minfi* was applied to normalize raw data. Common probes between the two experimental sets were then retained and annotated according to the Infinium HumanMethylation450 Beadchip annotation file. Methylation values were expressed as beta values (percentage of methylation, ranging from 0 to 1). Across the text and figures, DNA methylation is expressed as percentage ranging from 0 to 100%. Batch effects between the two experimental sets

were corrected using the ComBat function implemented in the *sva* R package (2) and, for the 7 samples replicated in the two experimental sets, mean beta values between the 450k and the EPIC experiment were calculated for each CpG probe. To predict the methylation of locus-specific repetitive elements (RE) we used the REMP bioconductor package (3).

Enrichment analysis was performed using Fisher exact test implemented in the R *stats* package. Heatmap and hierarchical clustering were performed using the *heatmap.2* function in R *gplots* package, with default settings (euclidean distance function, complete agglomeration method).

Differential methylation analysis

The analysis of differentially methylated regions (DMRs) indicated in Methods was applied only to the probes mapping in CpG-rich regions (CpG islands, shores and shelves) associated to a gene, according to the Infinium HumanMethylation450 Beadchip annotation. Multiple testing correction was performed using Benjamini–Hochberg procedure. To identify the DMRs that unambiguously distinguished the Progeroid and Non-Progeroid groups, we further filtered the list of significant (BH-corrected p-value <0.05) DMRs as follows: 1) for each DMR, we considered the sliding window of 3 adjacent probes having the smallest MANOVA p-value; 2) we performed k-means clustering on methylation values of the 3 probes, setting k=2 (corresponding to Progeroid and Non-Progeroid groups); 3) we retained only those DMRs in which all the CS samples were classified as Progeroid and all the WT and UVSS samples were classified as Non-Progeroid. It is worth to be noted that the same DMR can include both probes that gain and probes that lose methylation compared to controls. We assigned a DMRs as hypermethylated or hypomethylated by considering the direction of DNAm changes of the most significant (according to ANOVA) CpG probe within the DMR itself.

Functional enrichment analysis

The two biological samples were on one side the 7 Progeroid (CSI_A, CSI_B, and CSII) samples, and on the other side the 5 Non-Progeroid (WT + UVSS) samples. The specific implementation of GSEA for methylation datasets proposed by MethylGSA (4) was used. MethylGSA adapts the robust rank aggregation (RRA) approach to adjust for number of CpGs in DNA methylation gene set testing.

The input for the GSEA analysis is the list of adjusted p-values per probe issued from the differential methylation analysis at the MR and MP level (see above). For the MRs, a representative probe is considered per region, the one holding the minimum adjusted p-value, leading to an initial set

of 29603 probes. For MPs, instead, the initial set holds 44,1982 probes. Gene ontology terms (GO) with a minimum of 100 genes and a maximum of 500 genes associated were used to functionally score MRs.

For the visualization of GO terms associated with significant methylation changes in MRs and their corresponding genes (Fig. 2 and Fig. S5), a selection of terms and genes was done. The list of significant GO terms (GSEA adjusted p-value<0.1) was simplified by removing redundant terms with the REVIGO procedure (5) with default parameters and keeping only genes overlapping with genes associated to DMRs. R Scripts used for the above described analyses are available at: https://gitlab.pasteur.fr/cchica/methylage.

Epigenetic clocks analysis

Epigenetic age was calculated for different epigenetic clocks using the Horvath's online calculator (https://dnamage.genetics.ucla.edu/) and the pipeline described in Higgins-Chen et al. (6). For each clock, epigenetic age acceleration was calculated as the residuals of the regression of epigenetic age on chronological age. ANOVA was used to compare epigenetic age acceleration values between Progeroid and Non-Progeroid groups. GSE197724 was downloaded from GEO database.

RNA extraction and Real Time (RT)-qPCR

Total RNA of WT (911VI, 198VI), UVSS (UVSS1VI, UVSTA24) and CS (CS466VI, CS548VI, CS816VI, CS177VI) cells were isolated using the RNAeasy® Micro kit (Cat#74004; Qiagen) and mRNA were reverse transcribed with the SuperScript VI Reverse Transcriptase (Cat#18090050; Thermo Fisher Scientific). Resulting cDNAs were treated with RNAse H (Cat#2150B; Takara Bio) and quantified by RT-qPCR using the PowerUp™ SYBR™ Green Master mix (Cat#A25742; Thermo Fisher Scientific) on a StepOne Plus RealTime PCR system (Applied Biosystems). Data were analysed by the StepOne Plus RT PCR software v2.1 (Applied Biosystems) and normalized to TATA Binding Protein (TBP) mRNA level (=ΔCT). mRNA levels of each sample were calculated using the following formula; $2^{-\Delta CT}$, and plotted against their DNA methylation levels. Finally, the statistical test (two-tailed) and Pearson correlation were conducted using the GraphPad Prism v6.0 software (GraphPad software).

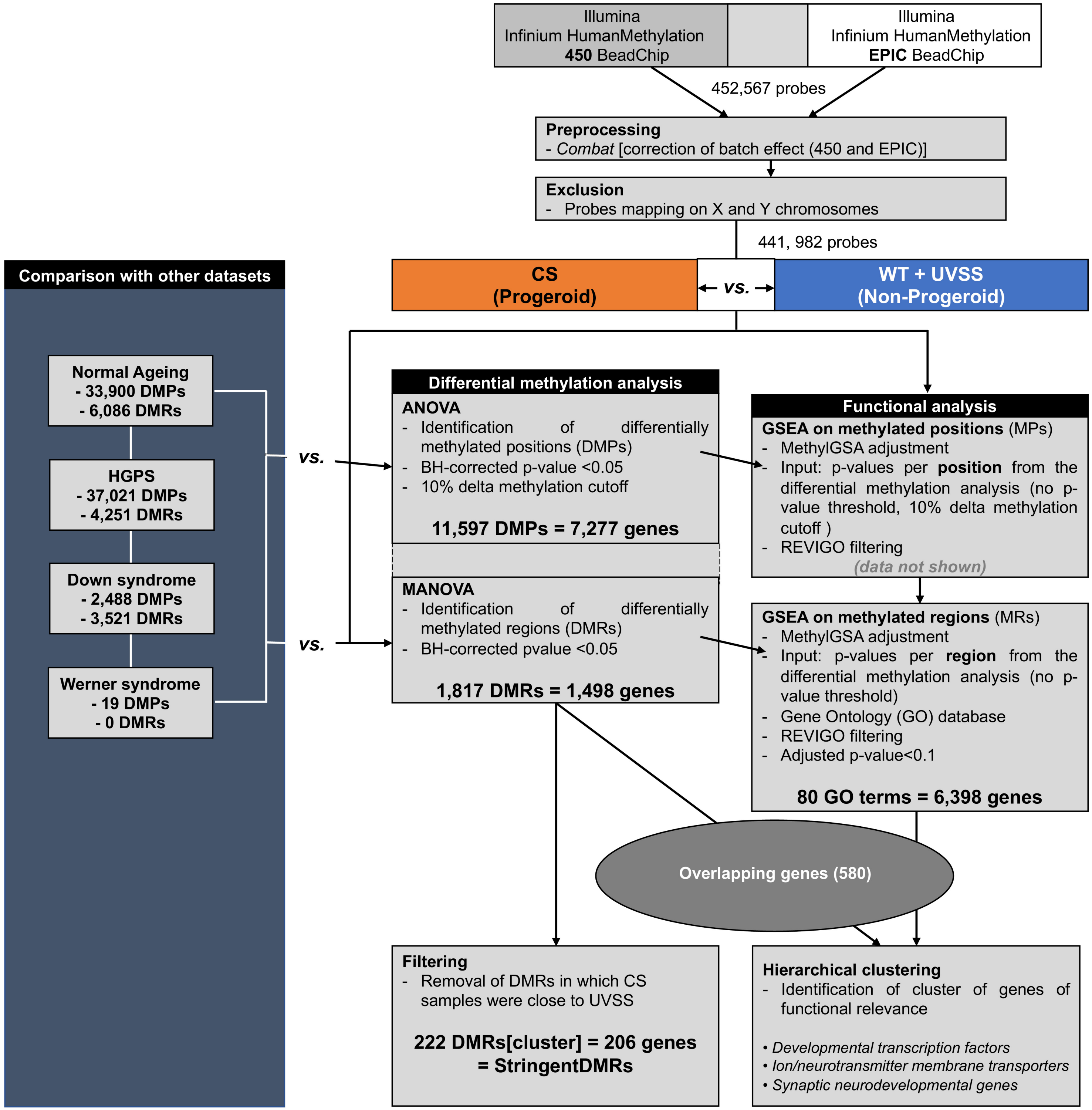

Figure S1. Analytic pipeline for the identification of the epigenomic signature of CS and common marks with pathophysiological ageing.
Flow chart of the pipeline used to identify CS-associated methylation positions and methylation regions, for the functional analysis and the comparison with other databases.

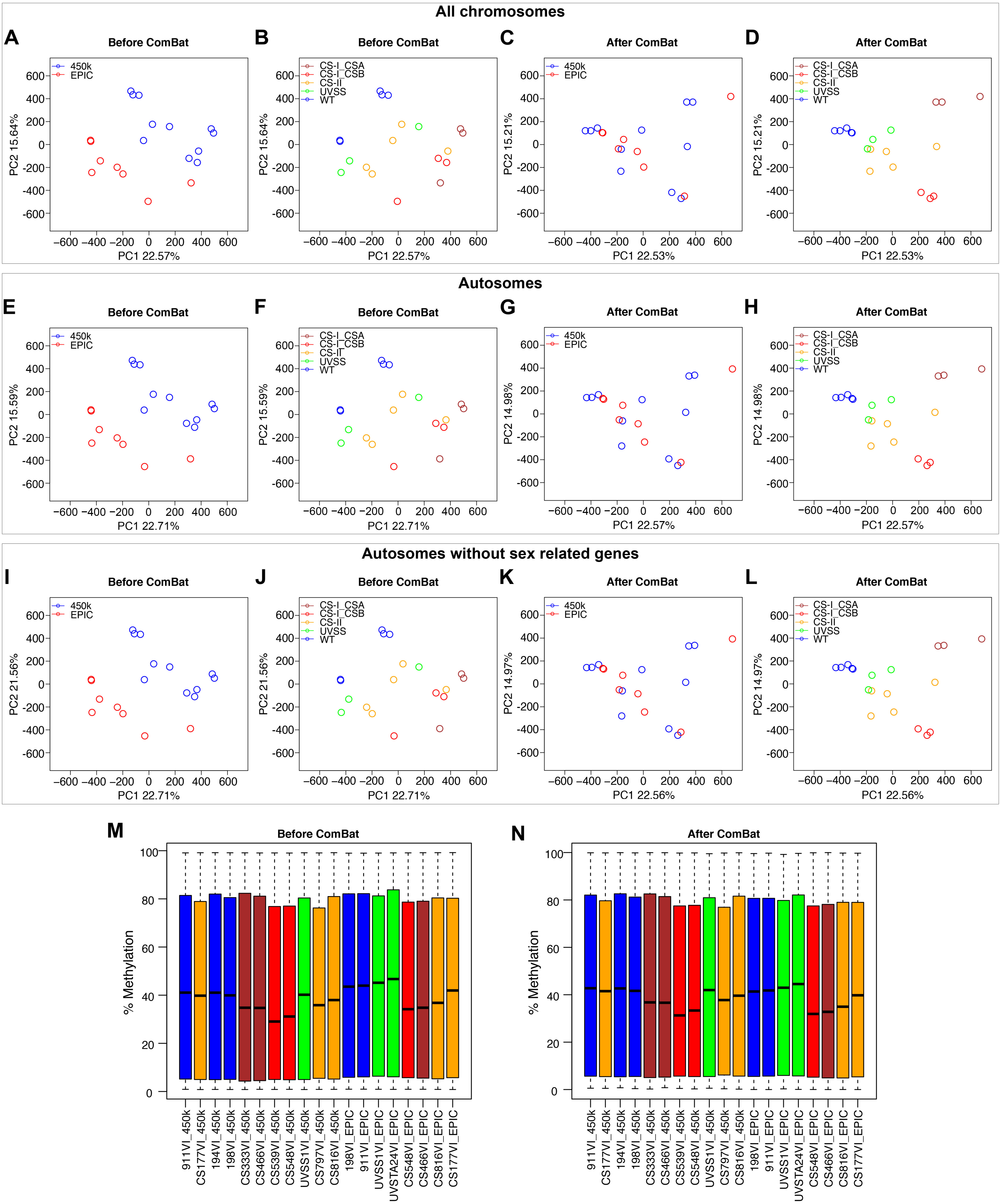

Figure S2. Effect of sex and Combat batch correction of the global DNA methylation pattern. PCA on methylation values of the 452567 probes common to the 450k and the EPIC arrays, before (**A, B**) and after (**C, D**) Combat algorithm to correct for the batch effect associated with the two platforms. In panels A and C, samples are colour labelled according to the microarray platform, and in B and D according to the disease group. (**E-H**) The same as in A-D, after removing the probes mapping on the X and Y chromosomes. (**I-L**) The same as in A-D, after removing the probes mapping on the X and Y chromosomes, and autosomic probes reported to be associated with sex. Boxplot of global percentage of DNAm in each sample used for the Infinium HumanMethylation450 Beadchip and the Infinium MethylationEPIC BeadChip microarrays, before (**M**) and after (**N**) batch correction.

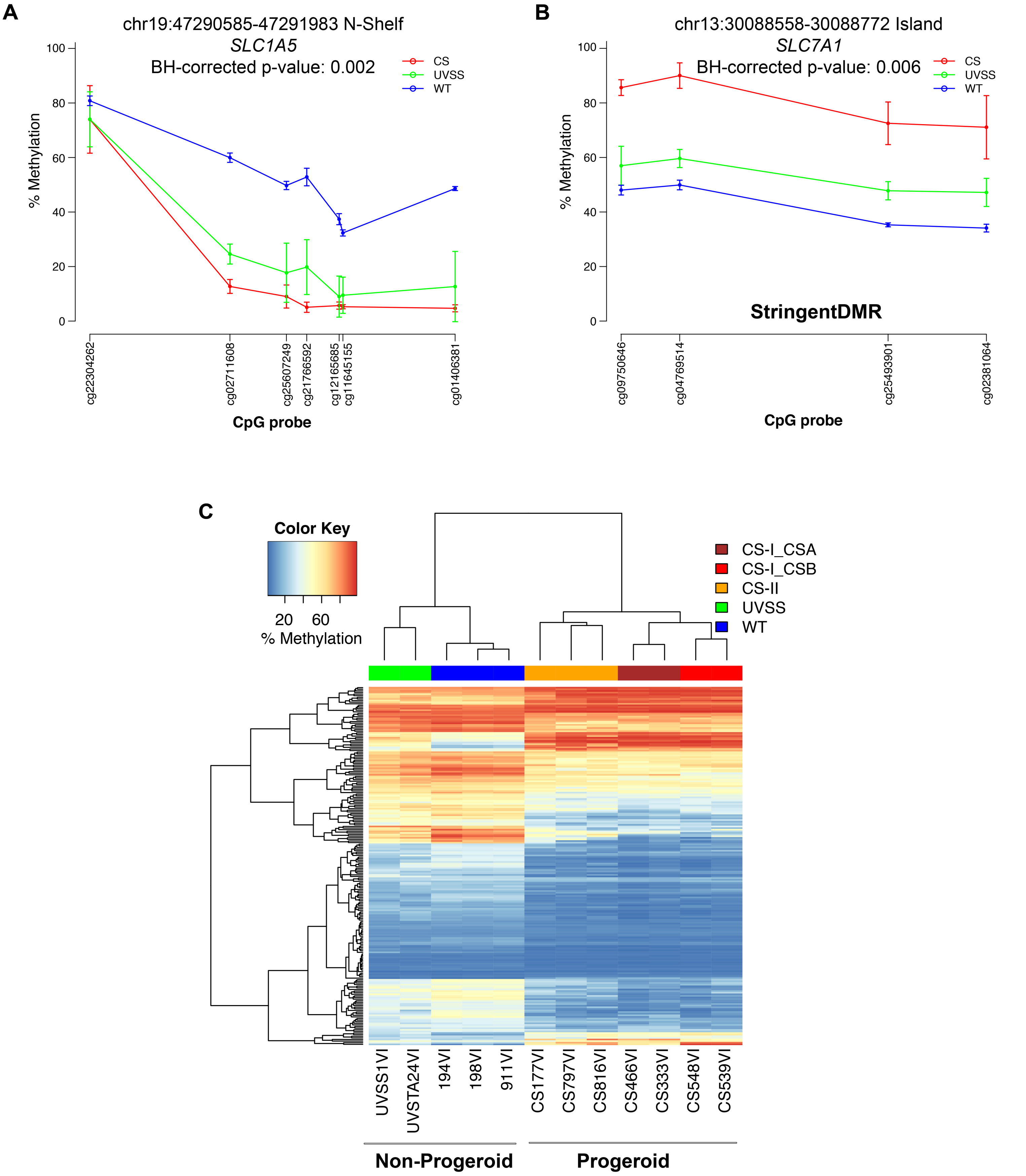

Figure S3. StringentDMRs.
Lineplots displaying the percentage of DNAm of each analysed CpG probe of WT, UVSS, and CS cells in the differentially methylated regions associated with (**A**) *SLC1A5*, and (**B**) *SLC7A1.* (A) is an example of a UVSS DNA methylation profile intermediary between WT and CS samples, whereas (B) is an example of DNA methylation profiles that unambiguously separate Progeroid (CS) from Non-Progeroid (WT + UVSS) groups, and is thereby part of the Stringent DMRs category. (**C**) The heatmap reports the percentage of DNAm for the 222 StringentDMRs identified in the Progeroid *versus* non-Progeroid condition (CpG probes are in rows, samples are in columns and are color-coded). Dendrograms depict hierarchical clustering of probes and samples.

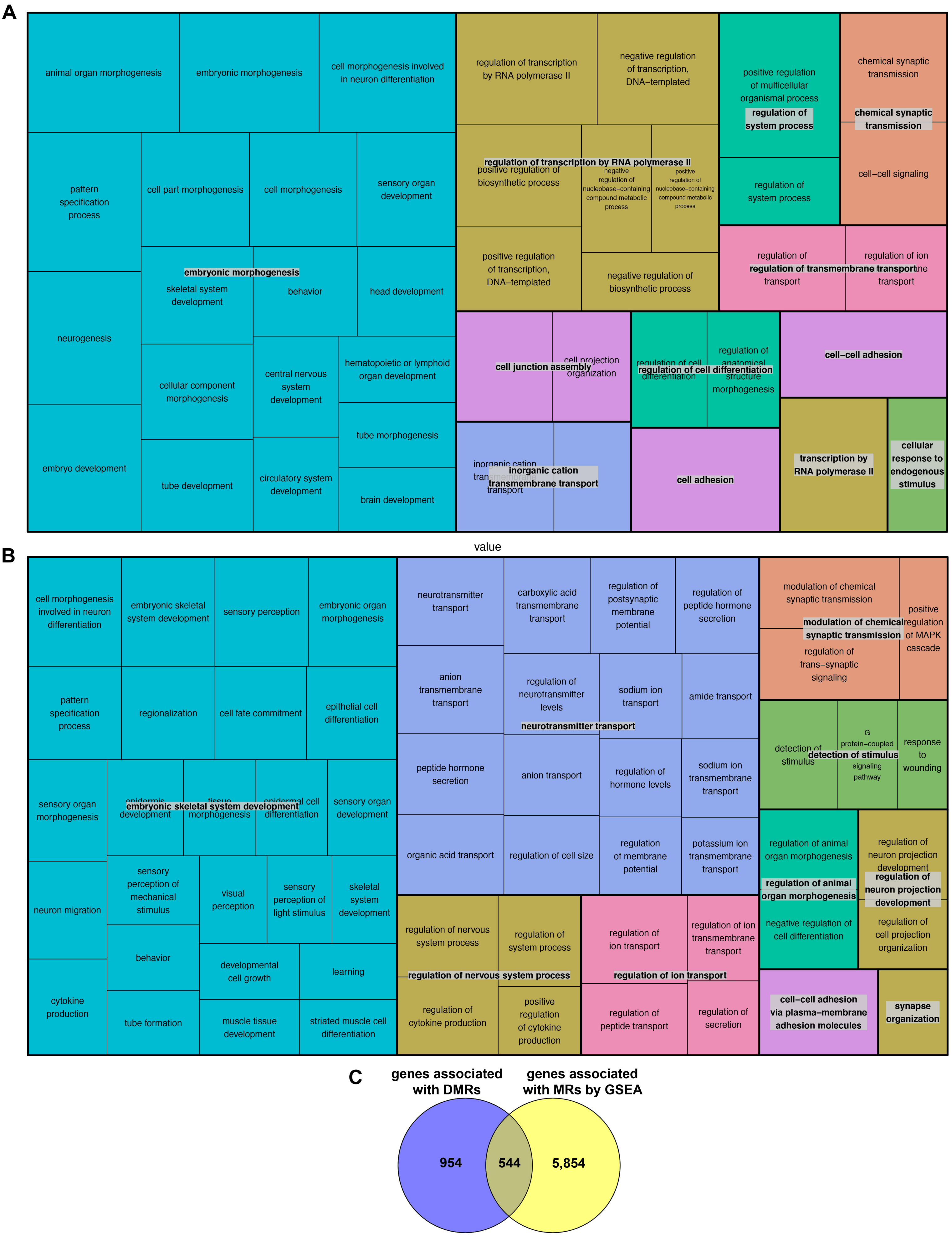

Figure S4. Hierarchical grouping of semantically related GO terms identified by GSEA. REVIGO treemap representing enriched GO terms (Biological Process) identified by GSEA analysis of (**A**) methylated positions, and (**B**) methylated regions. Semantically similar terms are grouped in clusters identified by a “meta-term“ (text with a grey background) and by a distinct color. Rectangle areas represent the -log10p value of each corresponding term. (**C**) Venn diagram representing the fraction of genes responsible for the GO term enrichment (544), out of the total list of genes associated with DMRs (1498), and out of the total number of genes identified by the GSEA analysis (6398).

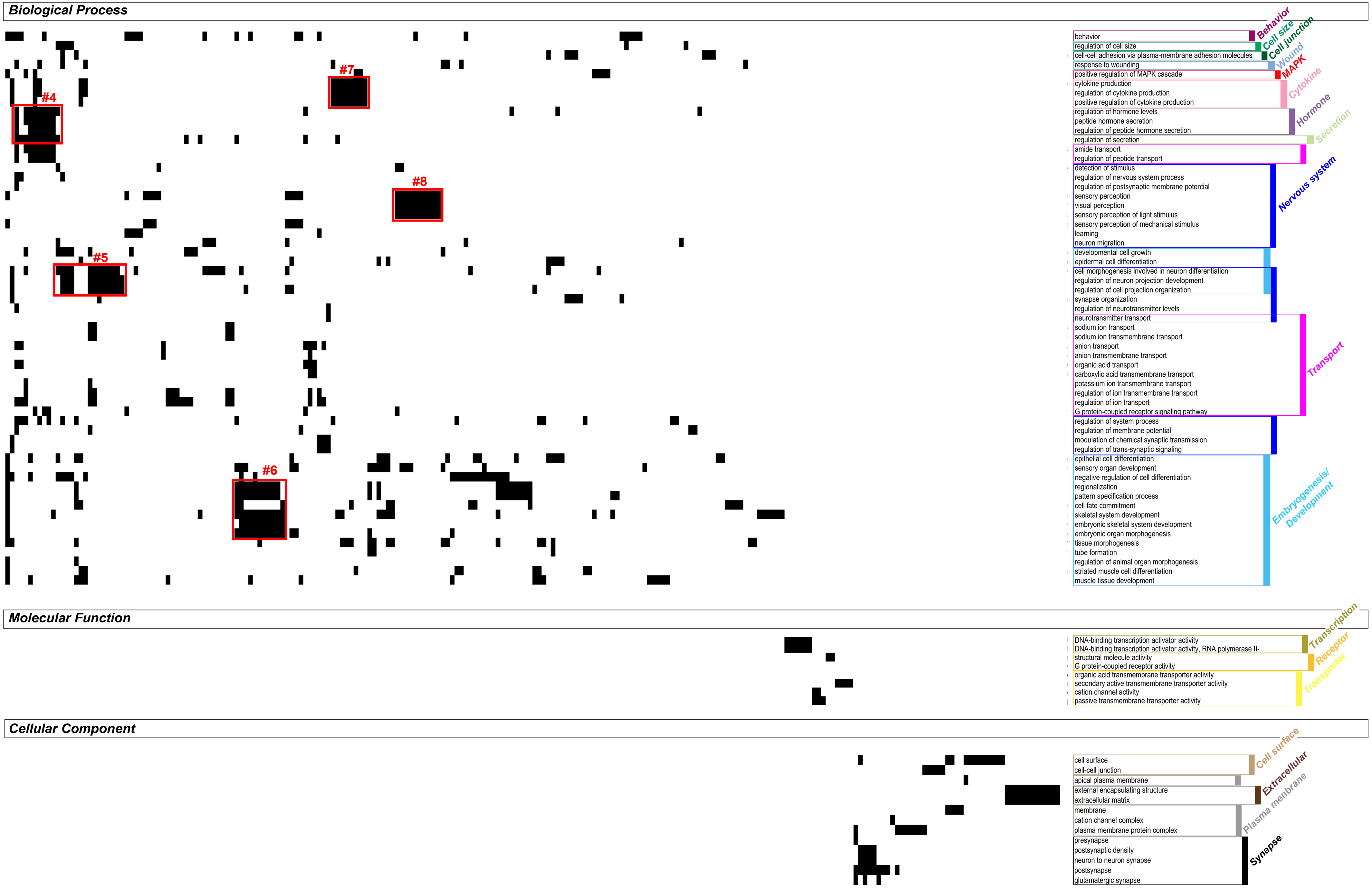

Figure S5. Representation of genes belonging to only one category of enriched GO terms identified in GSEA.
Heatmap plots reporting the relationship between the enriched GO terms identified in the Progeroid *vs.* Non-Progeroid GSEA analysis and the genes associated with DMRs belonging to these terms. Only genes specific to each category (Biological Process, Molecular Function, Cellular Component) are presented. The red frames indicate identified clusters of genes.

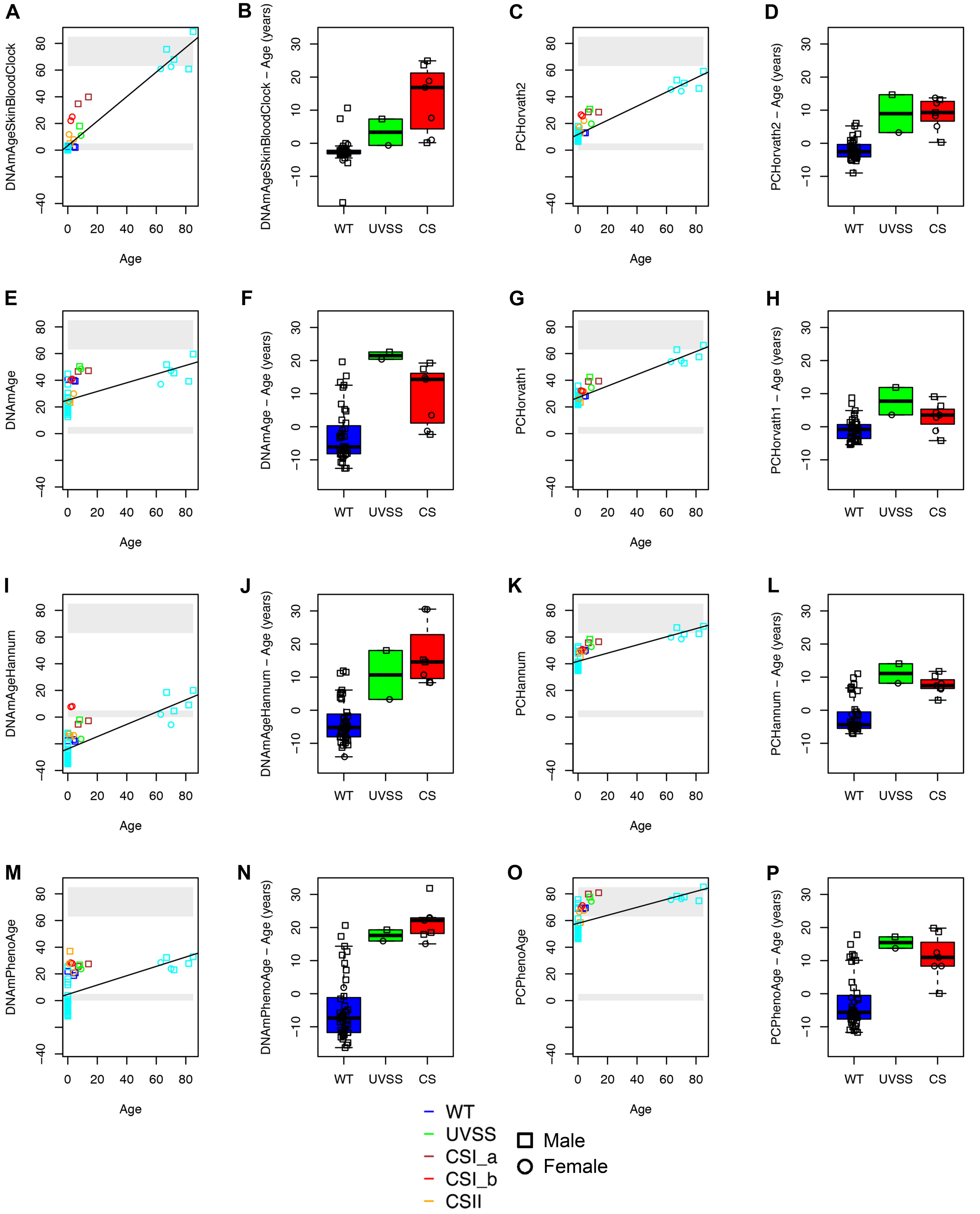

Figure S6. Epigenetic age acceleration according to different clocks and including samples from the GSE197724 dataset. For each clock, Skin&Blood (**A**), PCHorvath2 (**C**), Horvath pan-tissue (**E**), PCHorvath1 (**G**), Hannum (**I**) , PCHannum (**K**), PhenoAge (**M**), and PCPhenoAge (**O**) is reported the scatter plot of epigenetic age *vs* chronological age. The regions of the plots highlighted in grey correspond to the expected epigenetic age range of WT fibroblasts from the GSE197724 dataset, on the basis of their chronological age. The black line represents the regression of epigenetic age on chronological age. The corresponding bloxplots of epigenetic age acceleration values in WT, UVSS, and CS groups are shown in (**B**), (**D**), (**F**), (**J**), (**L**), (**N**), and (**P**).

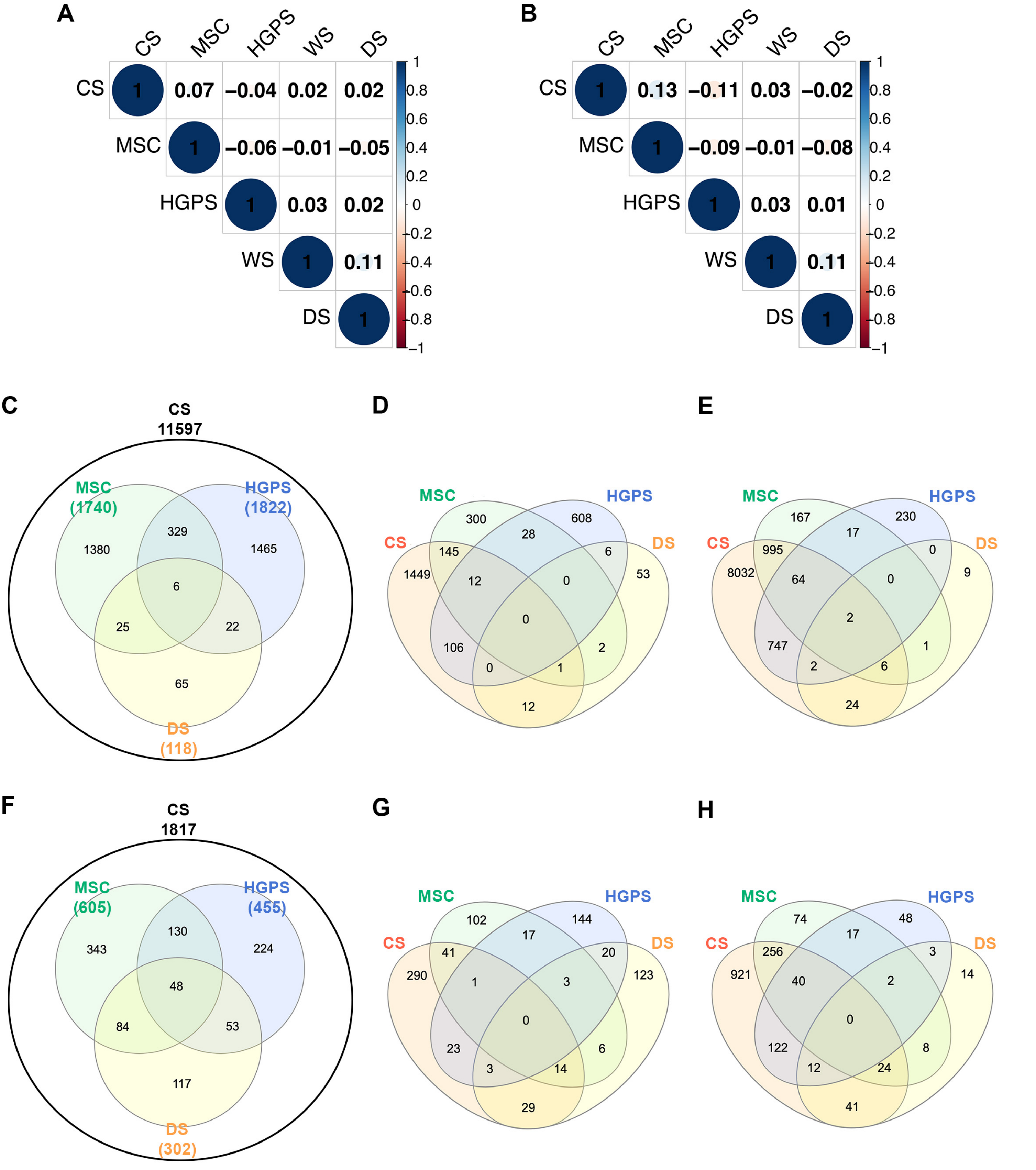

Figure S7: Comparison of DNAm changes in CS, in other progeroid diseases, and in normal aging. **(A)** Correlation matrix plot showing the magnitude of correlation between the DNAm changes observed in CS, HGPS, DS, WS and MSC datasets, considering the entire set of probes of the microarray (452567). **(B)** Correlation matrix plot showing the magnitude of correlation between the DNAm changes observed in CS, HGPS, DS, WS and MSC datasets, considering only the 11,597 CS-specific DMPs. **(C)** Venn diagram showing the number of common and specific DMPs in Normal Ageing, HGPS, and DS datasets, among the 11,597 DMRs identified in CS (represented with an external diagram). Common and specific hypo- **(D)** and hyper-methylated **(E)** DMRs in CS, Normal Ageing, HGPS, and DS datasets. **(F)** Venn diagram showing the number of common and specific DMRs in Normal Ageing, HGPS, and DS datasets, among the 1817 DMRs identified in CS (represented with an external diagram). Common and specific hypo- **(G)** and hyper-methylated **(H)** DMRs in CS, Normal Ageing, HGPS, and DS datasets.

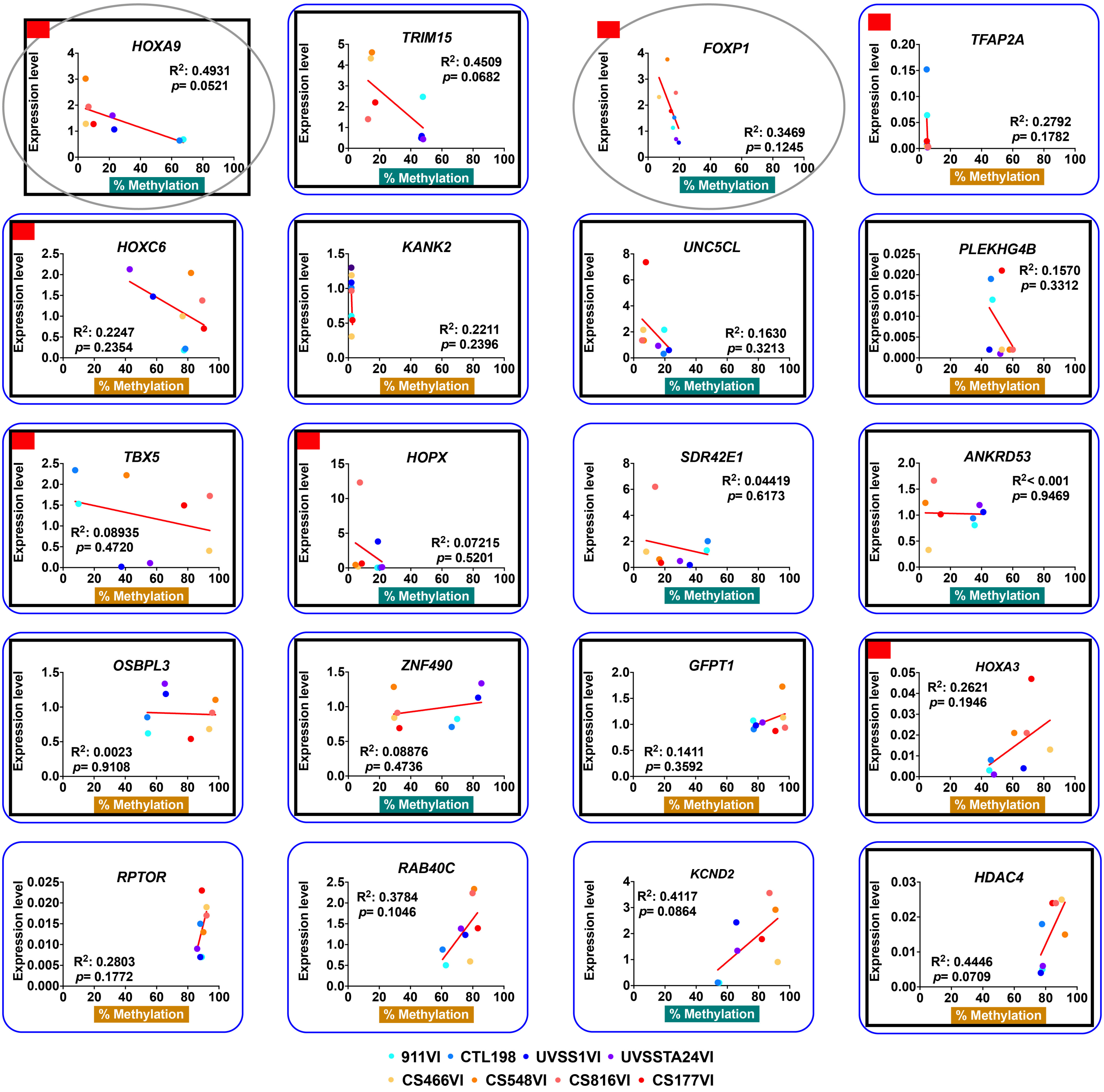

Figure S8. DNA methylation/transcription correlation in skin fibroblasts (remaining genes).
For each cell line of our dataset, the expression of selected genes was plotted against the percentage of DNAm. Out of the 31 gene tested, 11 displayed significant correlation and are shown in Fig. 4A. The remaining 20 genes did not show a significant correlation, and are shown here. Our of these 20 genes, 11 tended to be inversely correlated (*HOXA9, TRIM15, FOXP1, TFAP2A, HOXC6, KANK2, UNC5CL, PLEKHG4B*), 5 tended to be directly correlated (*HOXA3, RPTOR, RAB40C, KCDN2, HDAC4*), and 4 were not correlated (*ANKDR53, OSBPL3, ZNF490, GFPT1*). The linear regression curve is indicated in red. The Pearson's Correlation Coefficient was used to assess the correlation between methylation and transcription levels. The R squared (R^2) and *p-value* (*p*) are indicated in each graph. The direction of methylation change (hypermethylation and hypomethylation in the Progeroid *vs* Non-Progeroid group), is indicated with gold and green highlight of the X-axis label (% Methylation), respectively. Genes that showed differential methylation also in normal ageing and/or other progeroid diseases (14/20) are framed in black (rectangles). Genes present in the top list of DMR and/or StringentDMRs (18/20) are framed in dark blue (rounded rectangles). Genes selected because of their function (*HOXA9, FOXP1*) are framed in grey (ovals). TFs (7/20) are identified with a red square on the left upper corner. Gene selected for being differentally methylated specifically in CS (not in other datasets) are: *FOXP1, TFAP2A,SDR42E1, RPTOR, RAB40C, and KCND2*. These characteristics are not mutually exclusive.

Table S1. Top 50 hypo- and hyper-methylated DMPs of Progeroid *versus* Non-Progeroid condition upon single CpG analysis

Hypomethylated DMPs

Probe	CHR	MAPINFO	Gene	CpG island name	Relation with respect to the CpG island	Adjusted *P* value
cg04902529	1	4193806	-	chr1:4192633-4193243	S_Shore	**7.25E-05**
cg01074325	6	30138178	TRIM15	chr6:30139718-30140263	N_Shore	**7.53E-05**
cg10929628	13	112169571	-	chr13:112172232-112172441	N_Shelf	**7.53E-05**
cg18103548	10	102901943	-	chr10:102899822-102900263	S_Shore	**7.53E-05**
cg00406211	10	121077022	GRK5	-	-	**1.26E-04**
cg01293179	2	176996285	HOXD8	chr2:176993479-176995557	S_Shore	**1.26E-04**
cg07783843	2	176997311	-	chr2:176993479-176995557	S_Shore	**1.27E-04**
cg09634455	11	8337396	-	-	-	**1.73E-04**
cg15625495	1	151163316	VPS72	-	-	**1.73E-04**
cg15687395	5	79379309	-	-	-	**1.73E-04**
cg24852548	4	57522632	HOPX	chr4:57521621-57522703	Island	**2.05E-04**
cg04331756	10	8546532	-	-	-	**2.40E-04**
cg04737087	2	71205052	ANKRD53	chr2:71205563-71206529	N_Shore	**3.05E-04**
cg09222023	3	138669322	C3orf72	chr3:138668635-138669323	Island	**3.41E-04**
cg06162185	10	8546551	-	-	-	**4.57E-04**
cg07190012	11	16633647	-	chr11:16634957-16635969	N_Shore	**4.57E-04**
cg14446658	5	6845744	-	-	-	**4.57E-04**
cg14456683	3	147127010	ZIC1	chr3:147126988-147128999	Island	**4.57E-04**
cg17686487	5	50686281	ISL1	chr5:50685453-50686148	S_Shore	**4.57E-04**
cg23159970	12	2690385	CACNA1C	-	-	**4.57E-04**
cg11885357	3	138152902	ESYT3	chr3:138153269-138154621	N_Shore	**5.17E-04**
cg08079908	2	176997277	-	chr2:176993479-176995557	S_Shore	**6.62E-04**
cg04276953	1	18980700	PAX7	-	-	**7.12E-04**
cg10893095	6	85478296	-	-	-	**7.15E-04**
cg22130834	1	55267046	TTC22	chr1:55266277-55267058	Island	**7.15E-04**
cg26060817	5	14406676	TRIO	-	-	**7.69E-04**
cg01261503	17	62493599	POLG2	chr17:62492882-62493323	S_Shore	**7.74E-04**
cg02093811	2	233277840	-	chr2:233274080-233274525	S_Shelf	**7.74E-04**
cg03420881	2	130763516	-	chr2:130763483-130763764	Island	**7.74E-04**
cg04388792	19	12707977	ZNF490	chr19:12707696-12708114	Island	**7.74E-04**
cg10303978	16	50982437	-	-	-	**7.74E-04**
cg13501907	2	176997504	-	chr2:176993479-176995557	S_Shore	**7.74E-04**
cg13604154	9	129349764	-	-	-	**7.74E-04**
cg14162361	9	82449859	-	-	-	**7.74E-04**
cg19893664	14	105619634	JAG2	chr14:105622122-105622573	N_Shelf	**7.74E-04**
cg22891862	10	101279697	-	chr10:101279941-101280382	N_Shore	**7.74E-04**
cg26299756	6	85478807	-	chr6:85482569-85484718	N_Shelf	**7.74E-04**
cg08407620	6	24743861	-	-	-	**8.68E-04**
cg08893833	16	31045809	STX4	chr16:31044353-31045495	S_Shore	**8.68E-04**
cg07078114	9	139606202	FAM69B	chr9:139606300-139607433	N_Shore	**9.81E-04**
cg07973709	4	147164778	-	-	-	**1.03E-03**

cg23743573	11	89867420	NAALAD2	-	-	**1.05E-03**
cg05167857	16	86121574	-	-	-	**1.09E-03**
cg09190051	15	57026056	ZNF280D	chr15:57025347-57026150	Island	**1.09E-03**
cg14418226	6	40996092	UNC5CL	chr6:40995802-40996241	Island	**1.09E-03**
cg27301674	8	885217	-	-	-	**1.09E-03**
cg14612133	5	14406585	TRIO	-	-	**1.10E-03**
cg00195749	17	77764418	-	chr17:77763364-77767356	Island	**1.16E-03**
cg10925082	12	15114703	ARHGDIB	-	-	**1.16E-03**
cg18008037	7	158272029	PTPRN2	-	-	**1.16E-03**

Hypermethylated DMPs

**Probe**	**CHR**	**MAPINFO**	**Gene**	**CpG island name**	**Relation with respect to the CpG island**	**Adjusted *P* value**
cg00737846	4	183214573	-	-	-	**1.73E-04**
cg23894227	1	144814628	NBPF9	-	-	**3.52E-04**
cg01048107	8	72757924	MSC	chr8:72755783-72756667	S_Shore	**5.46E-04**
cg12779575	17	62208434	ERN1	chr17:62206971-62208050	S_Shore	**5.46E-04**
cg07244076	4	183084497	-	-	-	**7.74E-04**
cg08855449	3	147125962	ZIC1	chr3:147126988-147128999	N_Shore	**7.74E-04**
cg16655291	12	122012243	KDM2B	chr12:122016170-122017693	N_Shelf	**7.74E-04**
cg26426334	1	68403250	-	-	-	**7.74E-04**
cg16778405	16	653443	RAB40C	chr16:649465-650057	S_Shelf	**8.76E-04**
cg03256963	15	72105880	NR2E3	chr15:72104191-72104418	S_Shore	**9.36E-04**
cg09750646	13	30088615	SLC7A1	chr13:30088558-30088772	Island	**1.09E-03**
cg12419052	3	147126121	ZIC1	chr3:147126988-147128999	N_Shore	**1.15E-03**
cg00998744	3	147142198	-	chr3:147142181-147142391	Island	**1.16E-03**
cg07591442	2	69615105	GFPT1	chr2:69614119-69614616	S_Shore	**1.16E-03**
cg11664283	4	182909542	-	-	-	**1.16E-03**
cg16102778	6	30163098	TRIM26	-	-	**1.17E-03**
cg17783640	19	2188889	DOT1L	chr19:2190989-2191262	N_Shelf	**1.17E-03**
cg26021960	3	147140297	-	chr3:147142181-147142391	N_Shore	**1.25E-03**
cg06813862	12	89346059	-	-	-	**1.26E-03**
cg04769514	13	30088640	SLC7A1	chr13:30088558-30088772	Island	**1.34E-03**
cg24899806	7	119914282	KCND2	chr7:119915062-119915485	N_Shore	**1.62E-03**
cg03219658	12	114863053	-	-	-	**1.67E-03**
cg12404181	10	5688515	ASB13	-	-	**1.67E-03**
cg01305981	5	135266673	FBXL21	chr5:135265848-135266517	S_Shore	**1.67E-03**
cg17554126	12	114836794	TBX5	chr12:114838312-114838889	N_Shore	**1.74E-03**
cg06908052	17	78880396	RPTOR	chr17:78880030-78880278	S_Shore	**1.75E-03**
cg16240162	3	147106890	ZIC4	chr3:147108511-147111703	N_Shore	**1.87E-03**
cg12224131	2	161128123	-	chr2:161126364-161126825	S_Shore	**1.90E-03**
cg01914365	3	32511650	-	chr3:32509265-32509525	S_Shelf	**2.13E-03**
cg15806880	11	624603	MUPCDH	chr11:624780-625053	N_Shore	**2.16E-03**
cg07861448	8	72757787	MSC	chr8:72755783-72756667	S_Shore	**2.17E-03**
cg08799394	10	97049193	PDLIM1	chr10:97050185-97051124	N_Shore	**2.17E-03**
cg20676047	11	857071	TSPAN4	chr11:858575-858932	N_Shore	**2.17E-03**

cg18470456	13	96204493	CLDN10	chr13:96204691-96205496	N_Shore	**2.52E-03**
cg18000764	3	12947655	IQSEC1	chr3:12949699-12950025	N_Shelf	**2.56E-03**
cg06112335	7	119694495	-	-	-	**2.72E-03**
cg24260327	12	125023590	-	chr12:125023589-125023809	Island	**2.76E-03**
cg26764362	6	45451585	RUNX2	-	-	**2.86E-03**
cg18530511	3	159479909	SCHIP1	chr3:159480946-159483023	N_Shore	**3.11E-03**
cg19860752	18	37332566	LOC647946	-	-	**3.11E-03**
cg14890509	19	45943865	-	chr19:45942865-45943396	S_Shore	**3.15E-03**
cg13474918	7	119914578	KCND2	chr7:119915062-119915485	N_Shore	**3.44E-03**
cg01328621	15	68567276	-	chr15:68569974-68571058	N_Shelf	**3.48E-03**
cg13938349	7	44364084	CAMK2B	chr7:44364432-44365328	N_Shore	**3.49E-03**
cg17401780	10	129949004	-	chr10:129947728-129949099	Island	**3.49E-03**
cg17853801	17	21103363	TMEM11	-	-	**3.49E-03**
cg17021933	12	9063834	-	chr12:9066946-9067480	N_Shelf	**3.57E-03**
cg17324339	8	48656349	-	-	-	**3.86E-03**
cg16748008	7	27155002	HOXA3	chr7:27154999-27155426	Island	**4.02E-03**
cg09663836	12	133102331	FBRSL1	chr12:133102168-133102604	Island	**4.12E-03**

DMPs ($p<0.05$; absolute difference in mean DNAm values > 10%) are ranked according to the adjusted p-value. Mapping information on DMPs are given by the probe ID, the chromosome and base pair numbers, the CpG Island coordinate, the relation respect to the CpG island, and the associated gene (when applicable).

Table S4. Top 50 hypo- and hyper-methylated DMRs of Progeroid *versus* Non-Progeroid

Hypomethylated DMRs

CHR	CpG island name	Relation with respect to the CpG island	Gene(s)	Adjusted *P* value
**4**	**chr4:57521621-57522703**	**Island**	**HOPX**	**4.95E-04**
**6**	**chr6:40995802-40996241**	**Island**	**UNC5CL**	**1.14E-03**
16	chr16:88949975-88950247	Island	CBFA2T3	**1.32E-03**
**6**	**chr6:30139718-30140263**	**N_Shore**	**TRIM15**	**1.54E-03**
**1**	**chr1:3195376-3196052**	**N_Shelf**	**PRDM16**	**1.54E-03**
**19**	**chr19:12707696-12708114**	**Island**	**ZNF490**	**1.54E-03**
19	chr19:33622910-33623231	Island	WDR88	**1.83E-03**
12	chr12:130935712-130935963	S_Shore	RIMBP2	**1.83E-03**
**19**	**chr19:47290585-47291983**	**N_Shelf**	**SLC1A5**	**1.83E-03**
3	chr3:138153269-138154621	N_Shore	ESYT3	**1.83E-03**
7	chr7:150647991-150648918	N_Shore	KCNH2	**1.83E-03**
4	chr4:55991402-55992171	Island	KDR	**1.90E-03**
17	chr17:18128577-18128821	Island	LLGL1	**1.90E-03**
**2**	**chr2:71205563-71206529**	**N_Shore**	**ANKRD53**	**2.00E-03**
8	chr8:28928996-28929718	Island	KIF13B	**2.00E-03**
8	chr8:126010436-126011194	S_Shore	SQLE	**2.15E-03**
5	chr5:50683285-50683615	Island	ISL1	**2.75E-03**
5	chr5:154317459-154317930	S_Shore	GEMIN5	**2.75E-03**
3	chr3:138668635-138669323	Island	C3orf72	**2.91E-03**
12	chr12:49212162-49212642	N_Shore	CACNB3	**2.95E-03**
1	chr1:1098043-1100584	S_Shelf	MIR429	**3.04E-03**
1	chr1:34642382-34643024	Island	C1orf94	**3.04E-03**
21	chr21:46129391-46129689	S_Shore	C21orf29	**3.04E-03**
17	chr17:1465159-1466312	S_Shore	PITPNA	**3.04E-03**
12	chr12:52994772-52995312	N_Shore	KRT72	**3.04E-03**
**16**	**chr16:82044886-82045306**	**Island**	**SDR42E1**	**3.05E-03**
17	chr17:45500780-45501707	Island	C17orf57/ LOC100272146	**3.52E-03**
2	chr2:220377743-220377946	Island	ACCN4	**3.63E-03**
1	chr1:220960016-220960603	Island	MOSC1	**3.81E-03**
14	chr14:105622122-105622573	N_Shelf	JAG2	**3.95E-03**
1	chr1:18808873-18809667	N_Shelf	KLHDC7A	**4.07E-03**
17	chr17:78165077-78165293	N_Shelf	CARD14	**4.47E-03**
19	chr19:36365453-36365824	Island	APLP1	**4.77E-03**
3	chr3:44518838-44519368	S_Shore	ZNF445	**4.94E-03**
15	chr15:92459266-92459678	S_Shore	SLCO3A1	**4.99E-03**
6	chr6:31867691-31867957	N_Shore	EHMT2	**5.34E-03**
6	chr6:32975684-32975926	N_Shore	HLA-DOA	**5.34E-03**
6	chr6:24357719-24358309	Island	DCDC2/ KAAG1	**5.34E-03**
11	chr11:2949788-2951756	S_Shore	PHLDA2	**5.34E-03**
15	chr15:100346724-100347454	S_Shore	C15orf51	**5.34E-03**
22	chr22:43505951-43506167	Island	BIK	**5.34E-03**
5	chr5:1799461-1801905	Island	MRPL36/ NDUFS6	**5.36E-03**

1	chr1:24742671-24743039	Island	NIPAL3	**5.41E-03**
2	chr2:200335497-200336413	Island	FLJ32063	**5.63E-03**
16	chr16:1227260-1227476	Island	CACNA1H	**5.87E-03**
5	chr5:34915293-34916240	N_Shore	RAD1/BRIX1	**5.94E-03**
**4**	**chr4:57521621-57522703**	**S_Shore**	**HOPX**	**6.10E-03**
1	chr1:154300975-154301528	N_Shore	ATP8B2	**6.10E-03**
17	chr17:62492882-62493323	S_Shore	POLG2	**6.25E-03**
17	chr17:78444483-78444703	Island	NPTX1	**6.25E-03**

Hypermethylated DMRs

CHR	CpG island name	Relation with respect to the CpG island	Gene(s)	Adjusted *P* value
**7**	**chr7:25018999-25020255**	**N_Shore**	**OSBPL3**	**1.14E-03**
**12**	**chr12:114838312-114838889**	**N_Shore**	**TBX5**	**1.54E-03**
**3**	**chr3:147126988-147128999**	**N_Shore**	**ZIC1**	**1.83E-03**
**16**	**chr16:675906-676139**	**Island**	**RAB40C**	**1.83E-03**
**19**	**chr19:11307495-11308276**	**S_Shore**	**KANK2**	**1.87E-03**
17	chr17:1799541-1799756	S_Shelf	RPA1	**1.90E-03**
13	chr13:96204691-96205496	N_Shore	CLDN10	**1.90E-03**
6	chr6:6002471-6005125	N_Shore	NRN1	**2.91E-03**
**1**	**chr1:29213438-29214300**	**N_Shore**	**EPB41**	**2.95E-03**
**12**	**chr12:54408426-54408713**	**S_Shore**	**HOXC4/HOXC5/HOXC6**	**3.04E-03**
6	chr6:100912071-100913337	N_Shore	SIM1	**3.04E-03**
1	chr1:36771830-36773009	N_Shore	C1orf113	**3.52E-03**
**7**	**chr7:27154999-27155426**	**Island**	**HOXA3**	**4.20E-03**
19	chr19:14551998-14552255	N_Shore	PKN1	**4.43E-03**
**2**	**chr2:69614119-69614616**	**S_Shore**	**GFPT1**	**4.55E-03**
**7**	**chr7:119915062-119915485**	**N_Shore**	**KCND2**	**4.94E-03**
17	chr17:8126224-8126486	Island	C17orf44	**4.94E-03**
19	chr19:35633847-35634629	Island	FXYD7	**4.99E-03**
**13**	**chr13:30088558-30088772**	**Island**	**SLC7A1**	**5.63E-03**
15	chr15:41836145-41836612	S_Shore	RPAP1	**6.10E-03**
1	chr1:231003631-231004655	S_Shore	C1orf198	**6.40E-03**
5	chr5:135265848-135266517	S_Shore	FBXL21	**6.59E-03**
8	chr8:72755783-72756667	S_Shore	MSC	**7.40E-03**
14	chr14:74959680-74960907	N_Shore	ISCA2/ NPC2	**8.45E-03**
8	chr8:22089367-22089668	S_Shore	PHYHIP	**8.69E-03**
1	chr1:45189705-45190558	N_Shore	C1orf228	**9.03E-03**
19	chr19:8554998-8555251	Island	PRAM1	**9.03E-03**
**12**	**chr12:114833911-114834210**	**Island**	**TBX5**	**9.04E-03**
10	chr10:135083739-135083962	S_Shore	ADAM8	**9.33E-03**
17	chr17:80397431-80397642	S_Shore	HEXDC	**9.43E-03**
7	chr7:27143181-27143479	N_Shelf	HOXA2	**9.60E-03**
11	chr11:858575-858932	N_Shore	TSPAN4	**1.04E-02**
7	chr7:27227520-27229043	N_Shore	HOXA11A	**1.14E-02**
15	chr15:37172473-37173018	N_Shelf	LOC145845	**1.14E-02**

3	chr3:147108511-147111703	N_Shore	ZIC4	**1.15E-02**
10	chr10:134218268-134219615	S_Shelf	PWWP2B	**1.15E-02**
4	chr4:982814-984189	Island	IDUA/ SLC26A1	**1.15E-02**
1	chr1:55271665-55271972	N_Shore	C1orf177	**1.16E-02**
13	chr13:114849000-114852746	S_Shelf	RASA3	**1.18E-02**
1	chr1:1850098-1851391	S_Shore	TMEM52	**1.20E-02**
20	chr20:35374110-35374654	Island	NDRG3	**1.20E-02**
13	chr13:24152899-24154140	N_Shore	TNFRSF19	**1.22E-02**
12	chr12:133102168-133102604	Island	FBRSL1	**1.22E-02**
6	chr6:35453776-35454435	Island	TEAD3	**1.28E-02**
**17**	**chr17:78880030-78880278**	**S_Shore**	**RPTOR**	1.32E-02
13	chr13:113536815-113537308	S_Shore	ATP11A	**1.39E-02**
10	chr10:100993820-100994188	N_Shore	HPSE2	**1.41E-02**
5	chr5:133449320-133451260	S_Shore	TCF7	**1.43E-02**
12	chr12:108168986-108169570	N_Shore	ASCL4	**1.43E-02**
4	chr4:74486044-74486258	S_Shore	RASSF6	**1.43E-02**

DMRs are ranked according to the adjusted p-value. Mapping information on DMRs are given by the chromosome number, the CpG Island coordinate, the relation respect to the CpG island, and the associated gene. Lines in bold indicate DMRs and corresponding genes assessed for expression.

Table S6. Functional role in ageing of the top 20 hypo- and hyper-methylated StringentDMRs of Progeroid *versus* Non-Progeroid

Hypomethylated DMRs

CHR	CpG island name	Gene(s)	Description	Functional role in ageing	(Ref)
**4**	**chr4:57521621-57522703**	**HOPX**	**dTF**	- Ectopic expression of *HOPX* in human cells induces senescence through modulation of the MAPK and the Akt pathways<br>- Transgenic mice overexpressing *Hopx* develop severe cardiac hypertrophy, cardiac fibrosis (both age-related), and premature death<br>- *Hopx* knockout mice have a normal lifespan	(7, 8)
**19**	**chr19:12707696-12708114**	**ZNF490**	**TF**	/	
3	chr3:138153269-138154621	ESYT3	Transporter	- *Esyt3* knockout mice display normal life span	(9)
17	chr17:18128577-18128821	LLGL1	Cytoskeleton	/	
**2**	**chr2:71205563-71206529**	**ANKRD53**	**/**	/	
5	chr5:50683285-50683615	ISL1	dTF	- *Isl1* overexpression in mice minimize the hearing impairment that normally occurs with aging<br>- *Isl1* overexpression in mice induce a progressive age-related decline in hearing functions	(10, 11)
3	chr3:138668635-138669323	C3orf72	/	/	
1	chr1:1098043-1100584	MIR429	MicroRNA	- *MIR-429* knockdown in mice attenuates Aβ-induced cytotoxicity in cortical neurons<br>- Complete MIR-200 family knockdown (including *MIR-429*) induces cellular senescence in a human gastric cancer cell line	(12, 13)
1	chr1:34642382-34643024	C1orf94	/	/	
6	chr6:31867691-31867957	EHMT2	Methyltransferase	- Pharmacological inhibition of EHMT1/2 does not induce senescence in a human ovarian cancer cell line<br>- Pharmacological inhibition or knockdown of EHMT2 induce senescence in human fibroblasts and cancer cell lines<br>- Knockdown of *EHMT2* induces many hallmarks of ageing (in which model?)<br>- *EHMT2* overexpression attenuates induction of senescence in human cancer cell lines<br>- Knockout of *Ehmt2* in mice induces senescence in the pancreas	(14-18)
6	chr6:32975684-32975926	HLA DOA	HLA clas II	/	
5	chr5:1799461-1801905	MRPL36 /<br>NDUFS6	Mt Rb<br>Mt complex/I	- Null S. *cerevisae MRPL36* analog displays a decreased chronological lifespan<br>- Impaired *Ndufs6* (knockout or knockdown), that is associated with senescence induction, is an accelerator of adult stem cell ageing<br>- Replenishment of Ndufs6 in knockout mice rejuvenates senescent cells	(19, 20)
1	chr1:24742671-24743039	NIPAL3	Transporter	/	
5	chr5:34915293-34916240	RAD1/<br>BRIX1	DNA repair/<br>Rb biogenesis	- *RAD1* (alias *ERCC1*) mutation is responsible for a precocious ageing phenotype in the human and mouse	(21)
**4**	**chr4:57521621-57522703**	**HOPX**	**dTF**	*see above, HOPX*	
5	chr5:50685453-50686148	ISL1	dTF	*see above, ISL1*	
9	chr9:140033235-140034176	GRIN1	Transporter	- *Grin1* knockout mice show signs of neurodegeneration	(22, 23)

1	chr1:55266277-55267058	TTC22	/	/	
14	chr14:23859356-23859620	MYH6/<br>MIR208A	Ms contraction/<br>microRNA	/	
**11**	**chr11:77790478-77791101**	**NDUFC2**	**Mt complex I**	- *NDUFC2* is differentially expressed after treatment with senolytics that ameliorates age-dependent spinal disc degeneration in mice	(24)

Hypermethylated DMRs

CHR	CpG island name	Gene(s)	Description	Functional role in ageing (Ref)	
**12**	**chr12:114838312-114838889**	**TBX5**	**dTF**	- Mice expressing a particular Tbx5 variant develop a cardiac fibrosis in an age-dependent manner<br>- Ventricular (Heart) *Tbx5* knockout mice display reduced lifespan	(25, 26)
**16**	**chr16:675906-676139**	**RAB40C**	**/**	- *Rab40* null female flies display increased lifespan	(27)
**7**	**chr7:27154999-27155426**	**HOXA3**	**dTF**	**/**	
**2**	**chr2:69614119-69614616**	**GFPT1**	**Glucosamine**	**/**	
**7**	**chr7:119915062-119915485**	**KCND2**	**Transporter**	**/**	
17	chr17:8126224-8126486	C17orf44	LINCRNA	/	
**13**	**chr13:30088558-30088772**	**SLC7A1**	**Transporter**	**/**	
8	chr8:72755783-72756667	MSC	TF/dTF	/	
12	chr12:133102168-133102604	FBRSL1	/	/	
**17**	**chr17:78880030-78880278**	**RPTOR**	**mTOR regulation**	- Knockdown of *RPTOR* disrupts RAS-induced senescence or protect against senescence in human cells<br>- *Rptor* knockout mice show aging-dependent defects in ketogenesis<br>- Ectopic overexpression of *Rptor* in mice prevents aging-mediated reductions in PHLPP2 phosphatase levels<br>- Mutations in the gene encoding for raptor extend adult lifespan in *C. elegans*	(28-32)
13	chr13:113536815-113537308	ATP11A	Transporter	- Mice expressing a particular Atp11a variant display neuronal degeneration	(33)
7	chr7:27219309-27219750	HOXA10	dTF	- Knockdown of *HOXA10* enhances p53 acetylation and induces a cell cycle arrest in a human cancer cell line<br>- The senescence activator *p21* is a transcriptional target of HOXA10 in human cells	(34, 35)
6	chr6:43149736-43150009	CUL9	Ubiquitin ligase	- Genetic disruptions demonstrate that CUL9 controls senescence through p53 in human and mouse cells<br>- *Cul9* knockout mice have a normal lifespan	(36, 37)
**5**	**chr5:169578-169798**	**PLEKHG4B**	**/**	**/**	
**2**	**chr2:240153476-240153792**	**HDAC4**	**Deacetylase**	- Ectopic overexpression of *HDAC4* delays senescence in human fibroblasts<br>- *HDAC4* knockdown leads to premature senescence in human fibroblasts<br>- *HDAC4* knockdown in human and mouse cells triggers premature senescence<br>- *HDAC4* knockdown delays aggregate formation and rescues neuronal and functions in HD mouse models<br>- Silencing of HDAC4 homologs in *C. elegans* and Drosophila increases the lifespan, and specifically delays aging-associated physical declines in adult flies	(38-41)

**6**	**chr6:10411394-10413857**	**TFAP2A**	**TF/dTF**	-	TFAP2A is enriched at promoters of differentially expressed genes during replicative senescence	(42)
9	chr9:112081402-112082905	EPB41L4B	/		/	
1	chr1:17331885-17332093	ATP13A2	Transporter	-<br>-<br>-	*Atp13a2* knockout mice exhibit age-dependent sensorimotor dysfunctions and developed into aging-dependent phenotypes resembling those of autophagy impairment<br>*ATPA13A* null medaka fishes show a reduced lifespan<br>*ATPA13A* mutations are linked to Parkinson Disease	(43-47)
22	chr22:19753312-19755013	TBX1	dTF		/	
7	chr7:2774444-2774655	GNA12	Transducer		/	
5	chr5:145214649-145215139	PRELID2	/		/	

Mapping information on DMRs are given by the chromosome number, the CpG Island coordinate, and the associated gene. A description and the functional validated role in ageing (if any) of each gene is provided. Literature underscored in grey shows direct or inverse correlation with ageing-related phenotypes. Bold lines indicate StringentDMRs and corresponding genes assessed for expression. dTF: developmental transcription factor; TF: Transcription factor; Mt: mitochondrial; Rb: ribosome; Ms: Muscle. “/” is unknown.

Supplementary References

1. Aryee MJ*, et al.* (2014) Minfi: a flexible and comprehensive Bioconductor package for the analysis of Infinium DNA methylation microarrays. *Bioinformatics* 30(10):1363-1369.
2. Leek JT, Johnson WE, Parker HS, Jaffe AE, & Storey JD (2012) The sva package for removing batch effects and other unwanted variation in high-throughput experiments. *Bioinformatics* 28(6):882-883.
3. Zheng Y*, et al.* (2017) Prediction of genome-wide DNA methylation in repetitive elements. *Nucleic Acids Res* 45(15):8697-8711.
4. Ren X & Kuan PF (2019) methylGSA: a Bioconductor package and Shiny app for DNA methylation data length bias adjustment in gene set testing. *Bioinformatics* 35(11):1958-1959.
5. Supek F, Bosnjak M, Skunca N, & Smuc T (2011) REVIGO summarizes and visualizes long lists of gene ontology terms. *PLoS One* 6(7):e21800.
6. Higgins-Chen AT*, et al.* (2022) A computational solution for bolstering reliability of epigenetic clocks: Implications for clinical trials and longitudinal tracking. *Nat Aging* 2(7):644-661.
7. Chen Y, Yang L, Cui T, Pacyna-Gengelbach M, & Petersen I (2015) HOPX is methylated and exerts tumour-suppressive function through Ras-induced senescence in human lung cancer. *J Pathol* 235(3):397-407.
8. Kook H*, et al.* (2003) Cardiac hypertrophy and histone deacetylase-dependent transcriptional repression mediated by the atypical homeodomain protein Hop. *J Clin Invest* 112(6):863-871.
9. Herdman C, Tremblay MG, Mishra PK, & Moss T (2014) Loss of Extended Synaptotagmins ESyt2 and ESyt3 does not affect mouse development or viability, but in vitro cell migration and survival under stress are affected. *Cell Cycle* 13(16):2616-2625.
10. Huang M, Kantardzhieva A, Scheffer D, Liberman MC, & Chen ZY (2013) Hair cell overexpression of Islet1 reduces age-related and noise-induced hearing loss. *J Neurosci* 33(38):15086-15094.
11. Chumak T*, et al.* (2016) Deterioration of the Medial Olivocochlear Efferent System Accelerates Age-Related Hearing Loss in Pax2-Isl1 Transgenic Mice. *Mol Neurobiol* 53(4):2368-2383.
12. Fu S, Zhang J, & Zhang S (2018) Knockdown of miR-429 Attenuates Abeta-Induced Neuronal Damage by Targeting SOX2 and BCL2 in Mouse Cortical Neurons. *Neurochem Res* 43(12):2240-2251.
13. Yu L*, et al.* (2021) Complete loss of miR-200 family induces EMT associated cellular senescence in gastric cancer. *Oncogene*.
14. Watson ZL*, et al.* (2019) Histone methyltransferases EHMT1 and EHMT2 (GLP/G9A) maintain PARP inhibitor resistance in high-grade serous ovarian carcinoma. *Clin Epigenetics* 11(1):165.
15. Kondo Y*, et al.* (2008) Downregulation of histone H3 lysine 9 methyltransferase G9a induces centrosome disruption and chromosome instability in cancer cells. *PLoS One* 3(4):e2037.
16. Rao RA*, et al.* (2019) KMT1 family methyltransferases regulate heterochromatin-nuclear periphery tethering via histone and non-histone protein methylation. *EMBO Rep* 20(5).
17. Urrutia G*, et al.* (2021) Inactivation of the Euchromatic Histone-Lysine N-Methyltransferase 2 Pathway in Pancreatic Epithelial Cells Antagonizes Cancer Initiation and Pancreatitis-Associated

Promotion by Altering Growth and Immune Gene Expression Networks. *Front Cell Dev Biol* 9:681153.

18. Yuan Y*, et al.* (2012) A small-molecule probe of the histone methyltransferase G9a induces cellular senescence in pancreatic adenocarcinoma. *ACS Chem Biol* 7(7):1152-1157.
19. Marek A & Korona R (2013) Restricted pleiotropy facilitates mutational erosion of major life-history traits. *Evolution* 67(11):3077-3086.
20. Zhang Y*, et al.* (2020) Adult mesenchymal stem cell ageing interplays with depressed mitochondrial Ndufs6. *Cell Death Dis* 11(12):1075.
21. Gregg SQ, Robinson AR, & Niedernhofer LJ (2011) Physiological consequences of defects in ERCC1-XPF DNA repair endonuclease. *DNA Repair (Amst)* 10(7):781-791.
22. Intson K*, et al.* (2019) Progressive neuroanatomical changes caused by Grin1 loss-of-function mutation. *Neurobiol Dis* 132:104527.
23. Watanabe Y*, et al.* (2015) Age-Dependent Degeneration of Mature Dentate Gyrus Granule Cells Following NMDA Receptor Ablation. *Front Mol Neurosci* 8:87.
24. Novais EJ*, et al.* (2021) Long-term treatment with senolytic drugs Dasatinib and Quercetin ameliorates age-dependent intervertebral disc degeneration in mice. *Nat Commun* 12(1):5213.
25. Miyao N*, et al.* (2020) TBX5 R264K acts as a modifier to develop dilated cardiomyopathy in mice independently of T-box pathway. *PLoS One* 15(4):e0227393.
26. Rathjens FS*, et al.* (2021) Preclinical evidence for the therapeutic value of TBX5 normalization in arrhythmia control. *Cardiovasc Res* 117(8):1908-1922.
27. Duan X*, et al.* (2021) Regulation of lipid homeostasis by the TBC protein dTBC1D22 via modulation of the small GTPase Rab40 to facilitate lipophagy. *Cell Rep* 36(9):109541.
28. Ito M*, et al.* (2017) Selective interference of mTORC1/RAPTOR protects against human disc cellular apoptosis, senescence, and extracellular matrix catabolism with Akt and autophagy induction. *Osteoarthritis Cartilage* 25(12):2134-2146.
29. Jia K, Chen D, & Riddle DL (2004) The TOR pathway interacts with the insulin signaling pathway to regulate C. elegans larval development, metabolism and life span. *Development* 131(16):3897-3906.
30. Kim K*, et al.* (2016) mTORC1-independent Raptor prevents hepatic steatosis by stabilizing PHLPP2. *Nat Commun* 7:10255.
31. Kolesnichenko M, Hong L, Liao R, Vogt PK, & Sun P (2012) Attenuation of TORC1 signaling delays replicative and oncogenic RAS-induced senescence. *Cell Cycle* 11(12):2391-2401.
32. Sengupta S, Peterson TR, Laplante M, Oh S, & Sabatini DM (2010) mTORC1 controls fasting-induced ketogenesis and its modulation by ageing. *Nature* 468(7327):1100-1104.
33. Segawa K*, et al.* (2021) A sublethal ATP11A mutation associated with neurological deterioration causes aberrant phosphatidylcholine flipping in plasma membranes. *J Clin Invest* 131(18).
34. Bromleigh VC & Freedman LP (2000) p21 is a transcriptional target of HOXA10 in differentiating myelomonocytic cells. *Genes Dev* 14(20):2581-2586.
35. Zhang Y*, et al.* (2019) HOXA10 knockdown inhibits proliferation, induces cell cycle arrest and apoptosis in hepatocellular carcinoma cells through HDAC1. *Cancer Manag Res* 11:7065-7076.

36. Hollville E, *et al.* (2020) Characterization of a Cul9-Parkin double knockout mouse model for Parkinson's disease. *Sci Rep* 10(1):16886.
37. Li Z & Xiong Y (2017) Cytoplasmic E3 ubiquitin ligase CUL9 controls cell proliferation, senescence, apoptosis and genome integrity through p53. *Oncogene* 36(36):5212-5218.
38. Di Giorgio E, *et al.* (2021) HDAC4 degradation during senescence unleashes an epigenetic program driven by AP-1/p300 at selected enhancers and super-enhancers. *Genome Biol* 22(1):129.
39. Han X, *et al.* (2016) HDAC4 stabilizes SIRT1 via sumoylation SIRT1 to delay cellular senescence. *Clin Exp Pharmacol Physiol* 43(1):41-46.
40. Mielcarek M, *et al.* (2013) HDAC4 reduction: a novel therapeutic strategy to target cytoplasmic huntingtin and ameliorate neurodegeneration. *PLoS Biol* 11(11):e1001717.
41. Yu R, *et al.* (2021) Inactivating histone deacetylase HDA promotes longevity by mobilizing trehalose metabolism. *Nat Commun* 12(1):1981.
42. Hanzelmann S, *et al.* (2015) Replicative senescence is associated with nuclear reorganization and with DNA methylation at specific transcription factor binding sites. *Clin Epigenetics* 7:19.
43. Dehay B, *et al.* (2012) Loss of P-type ATPase ATP13A2/PARK9 function induces general lysosomal deficiency and leads to Parkinson disease neurodegeneration. *Proc Natl Acad Sci U S A* 109(24):9611-9616.
44. Kett LR, *et al.* (2015) alpha-Synuclein-independent histopathological and motor deficits in mice lacking the endolysosomal Parkinsonism protein Atp13a2. *J Neurosci* 35(14):5724-5742.
45. Matsui H, *et al.* (2013) ATP13A2 deficiency induces a decrease in cathepsin D activity, fingerprint-like inclusion body formation, and selective degeneration of dopaminergic neurons. *FEBS Lett* 587(9):1316-1325.
46. Schultheis PJ, *et al.* (2013) Atp13a2-deficient mice exhibit neuronal ceroid lipofuscinosis, limited alpha-synuclein accumulation and age-dependent sensorimotor deficits. *Hum Mol Genet* 22(10):2067-2082.
47. Wang R, *et al.* (2019) ATP13A2 facilitates HDAC6 recruitment to lysosome to promote autophagosome-lysosome fusion. *J Cell Biol* 218(1):267-284.
